# Chitosan-Based Nanocomposite Polymeric Membranes for Water Purification—A Review

**DOI:** 10.3390/ma14092091

**Published:** 2021-04-21

**Authors:** Angela Spoială, Cornelia-Ioana Ilie, Denisa Ficai, Anton Ficai, Ecaterina Andronescu

**Affiliations:** 1 Department of Science and Engineering of Oxide Materials and Nanomaterials, Faculty of Applied Chemistry and Materials Science, University Politehnica of Bucharest, 1-7 Gh Polizu Street, 011061 Bucharest, Romania; angela.spoiala83@yahoo.com (A.S.); cornelia_ioana.ilie@upb.ro (C.-I.I.); ecaterina.andronescu@upb.ro (E.A.); 2 Department of Inorganic Chemistry, Physical Chemistry and Electrochemistry, Faculty of Applied Chemistry and Materials Science, University Politehnica of Bucharest, 1-7 Gh Polizu Street, 050054 Bucharest, Romania; denisaficai@yahoo.ro; 3 National Centre for Micro and Nanomaterials and National Centre for Food Safety, Faculty of Applied Chemistry and Materials Science, University Politehnica of Bucharest, Spl. Independentei 313, 060042 Bucharest, Romania; 4 Academy of Romanian Scientists, 3 Ilfov Street, 050045 Bucharest, Romania

**Keywords:** chitosan, nanoparticles, nanocomposite polymeric membranes, water purification

## Abstract

During the past few years, researchers have focused their attention on developing innovative nanocomposite polymeric membranes with applications in water purification. Natural and synthetic polymers were considered, and it was proven that chitosan-based materials presented important features. This review presents an overview regarding diverse materials used in developing innovative chitosan-based nanocomposite polymeric membranes for water purification. The first part of the review presents a detailed introduction about chitosan, highlighting the fact that is a biocompatible, biodegradable, low-cost, nontoxic biopolymer, having unique structure and interesting properties, and also antibacterial and antioxidant activities, reasons for using it in water treatment applications. To use chitosan-based materials for developing nanocomposite polymeric membranes for wastewater purification applications must enhance their performance by using different materials. In the second part of the review, the performance’s features will be presented as a consequence of adding different nanoparticles, also showing the effect that those nanoparticles could bring on other polymeric membranes. Among these features, pollutant’s retention and enhancing thermo-mechanical properties will be mentioned. The focus of the third section of the review will illustrate chitosan-based nanocomposite as polymeric membranes for water purification. Over the last few years, researchers have demonstrated that adsorbent nanocomposite polymeric membranes are powerful, important, and potential instruments in separation or removal of pollutants, such as heavy metals, dyes, and other toxic compounds presented in water systems. Lastly, we conclude this review with a summary of the most important applications of chitosan-based nanocomposite polymeric membranes and their perspectives in water purification.

## 1. Chitosan: An Introduction

This paper describes the state-of-the-art perspectives of chitosan, and chitosan-based nanomaterials, for developing nanocomposite polymeric membranes for water purification. Water is the most important natural resource of the planet Earth, and its purity is vital for humans. Currently, the entire world is facing the most challenging issue, water contamination with different pollutants. Researchers and scientists are trying to overcome this emerging problem by seeking new materials with great adsorbent properties [1]. The review offers a detailed background of chitosan, mainly based on its unique properties and structure, and presents a multitude of examples of applications regarding chitosan-based nanocomposite as polymeric membranes in water treatment.

Among the biodegradable biopolymers, the ones based on polysaccharides such as chitosan have presented interest since the 19th century, when Rouget [2,3] showed the first chitin polymer. Chitosan is the most bountiful natural polysaccharide after cellulose and is produced by the deacetylation of chitin. The reason polysaccharides such as chitosan attracted special attention was because of their availability in nature, bio-renewability, sustainability, and having low-cost and impressive performance compared to other materials used in water treatment [4]. Chitosan is a biocompatible, biodegradable, bio-renewable, and non-toxic biopolymer, which is commercially derivates from shrimps and other sea crustaceans [5,6].

Chitosan presents very interesting polymeric forms, having unique structures and properties, and functionalities that target multiple applications from biomedicine, agricultural, and environmental protection [7,8]. Even though chitin has extraordinary properties and mechanics, it still has limitations [9,10,11], and because of that, the attention shifted towards chitosan [9,12]. 

Chitin conversion in chitosan uses chemical methods and enzymatic processes [13], but for better use in biomedical applications, chitosan is more appropriate due to better solubility in organic media and also water [14]. Being a copolymer, chitosan is formed by a linear β-(1→4) glycosidic link, very similar to the cellulose system having its structure of 2-acetamido-D-glucose and 2-amino-D-glucose combined through a glycosidic bond as shown in [15].

Chitosan is a product of chitin deacetylation when acetate is removed [16,17]. It proves that the molecular weight and degree of deacetylation influence the physical and chemical properties of chitosan. Having interesting composition and structure, and also unique physicochemical properties, chitosan provides plenteously of prospects through physical and chemical approaches to expand the applications in the removal of contaminants from water sources. Chitosan, being an important adsorbent material, presents great usage for the removal of toxins, such as microorganisms, heavy metals, dyes, and phenols, from drinking or wastewater. This review also presents the adsorption mechanisms and important factors which affect the adsorption process of chitosan-based polymeric membranes in the removal of pollutants from water sources [4,18]. They have shown that the modification of chitosan is crucial in improving the adsorbent capacity of the formed-membranes and expanding the applications towards the removal of contaminants from water. 

The tremendous importance of the adsorption mechanisms of chitosan and chitosan-based nanocomposite polymeric membranes is fundamental in offering better understandings that can enhance the performance of the membranes for the removal of pollutants [19]. The adsorption mechanism of chitosan as an adsorbent, especially for heavy metal removal, is very poorly studied. By understanding the critical role of the interaction between the amine group of the metal ions and the hydroxyl group from the chitosan, could bring better contribute towards a greater sorption property of the membrane for the removal of heavy metals. Normally, heavy metal adsorption through a modified chitosan surface can be measured by the electrostatic or chemical interaction (chelation or complexation), ion exchange, and nonpolar interaction such as van der Waals forces. The interaction mechanism of chitosan or chitosan-based materials with different metals affects the presence of diverse functional groups which can be characterized using various surface analyses such as X-ray photoelectron spectroscopy (XPS), X-ray diffractions (XRD), scanning electron microscopy (SEM), Fourier-transform infrared (FTIR) spectroscopy, and electron dispersive X-ray spectroscopy (EDX) [20]. 

There was a study made by Kuang et al. [21] that used the FTIR technique, and the data presented showed the complexation due to the grafting of magnetic chitosan with triethylenetetramine influenced the Pb(II) adsorption. In other research, Kwok et al. [22] described the removal of arsenic by using chitosan and nano chitosan where the adsorption mechanism, as electrostatic forces between arsenic oxyanions and chitosan-amine groups, were involved in the arsenic removal. Realizing that the presence of the metal could highly be absorbed on the chitosan surface through the van der Waals forces. 

Kumar et al. [23] reported chitosan-functionalized with graphene oxide as an excellent adsorbent for As(V) adsorption. It has been proven the regeneration capacity of the adsorbent by using NaOH. Moreover, the adsorbent regeneration and stability for three adsorption–desorption effective cycles, makes it an interesting adsorbent material. The authors claimed that the adsorption mechanisms were validated by the FTIR and XRD analyses and also through the EDS and XPS analysis.

Chitosan and its derivatives possess interesting characteristics with applicability in diverse fields of research, such as water treatment [24,25], food industry [26,27], cosmetics [28], agriculture [29], and biomedicine [30,31]. Because of the antibacterial and antioxidant properties of chitosan, it presents great potential in producing films, nanofibers, and hydrogels, which have great benefits in biomedical applications, also [32,33]. As stated above, chitosan exhibits many unique features and properties; nevertheless, its applications include various fields, which involve the development of wastewater membranes in treatment processes. The excellent antimicrobial activity of chitosan has received great importance in using it in promising technologies for industrial desalination. Wang et al. [34] studied the effects of surface wettability properties and fouling behavior of membrane distillation. A study reported that Forward Osmosis (FO) has great potential in the desalination of seawater and has low fouling capacity. Authors described the efficiency of FO using chitosan/GO membranes and the results proved the fouling resistance [35] and another study found that antibacterial performance against *E. coli* was better because of the cell membrane destruction [36]. Two studies in 2015 proved that the use of ZnO nanoparticles in chitosan polymeric membranes improves the antibacterial properties. Motshekga et al. [37] synthesized chitosan-based nanocomposite with bentonite-supported silver and ZnO nanoparticles to disinfect water. In another study made by Kamal et al. [38], they examined raw chitosan/ZnO composite coated on microfibrillar cellulose. Both studies showed excellent performance of the nanocomposite membranes against *E. coli*. 

Even if chitosan presents great qualities, such as low toxicity and high biodegradability, it still needs to overcome some other limitations regarding porosity, thermo-mechanical properties, and stability [19,39]. To pass these setbacks displayed by chitosan, researchers have devoted their efforts in developing different physicochemical modification methods that include functionalization through different chemical modifications of the polymer, which modified its properties for specific applications in various fields [40]. In addition to the forms of chitosan already mentioned above, researchers have succeeded in producing many more (such as gel beads, membranes, fibers, powders, sponges, hollow fibers, and also nanoparticles) [41,42,43,44].

It has been discovered that when suffering various chemical modifications, it enhances its flexibility and stability. After studying the commonly used modification methods that enhanced the functionalization, it was found that the mechanical strength of chitosan improved. It was another important aspect that enhanced the functionalization of chitosan, which involves using glyoxal, glutaraldehyde, or epichlorohydrin to reinforce the structure of chitosan through cross-linking [45,46,47,48]. Raw and unmodified chitosan has been used for the removal of diverse pollutants from the water as presented in the next study. Chitosan and chitosan cross-linked with glutaraldehyde (GLA-chitosan), epichlorohydrin (ECH-chitosan), and ethylene glycol diglycidyl ether (EGDE-chitosan) were examined for Cu(II) removal from water. The authors mentioned that the adsorption capacity of raw chitosan was greater than the cross-linked chitosan samples. Moreover, it has been stated that the adsorbents based on chitosan present the ability to be reused and regenerated [49].

In this review, we present a comprehensive and detailed view of the chitosan, highlighting its unique structure and interesting properties, and also the antibacterial and antioxidant activities, reasons for its use in developing polymeric membranes for water purification. The second part of the review focuses our attention on evaluating the effect of diverse nanoparticles on several polymeric membranes. Further, insights into understanding how various factors affect the adsorption performance of chitosan-based membranes are discussed. Finally, the review presents a summary regarding promising applications and perspectives of chitosan-based polymeric membranes in the environmental field.

## 2. Effect of Nanomaterials on Polymeric Membranes

In this section, will be presented the effect of diverse 0–3D nanomaterials that could impact developing polymeric membranes for applications in water treatment. Special attention will be brought to nanostructured materials, which are typically classified based on their dimensions, such as 0D-nanoparticles (Ag, TiO_2_, ZnO, and Cu); 1D-nanotubes (CNTs), nanofibers; 2D-nanosheets (graphene oxide, graphene, etc.); and 3D-bulk structures, which represents the number of dimensions that are not at nanoscale. However, bulk nanomaterials still exhibit features at nanoscale and can contain dispersions of nanoparticles, nanofibers, and nanotubes, as well as multilayered nanostructures.

Scanning the literature, there are many works that has used different nanomaterials, like silver (Ag) [50], titanium dioxide (TiO_2_) [51], zinc oxide (ZnO) [52], copper oxide (CuO) [53], alumina (Al_2_O_3_) [54], silica (SiO_2_) [55], magnetite (Fe_3_O_4_) [56], cobalt (Co) [57], zirconia (ZrO_2_) [58], carbon nanotubes (CNTs) [59], graphene oxide (GO) [60], and zeolites (such as, for instance, NaX) [61], under the evolution of nanocomposite polymeric membranes for water treatment applications (Table 1). There have been some innovative nanocomposite membranes commercialized for a multitude of segregation applications. Especially membranes having silver-based nanoparticles aimed for Reverse Osmosis (RO), as described by Sterlitech, and for water filtration by Lenntech and LG Chem (NanoH_2_O composite membranes) [50,62]. The components of nanocomposite polymeric membranes will be tested for water treatment at an industrial scale, which can be applied in the function of its high selectivity, self-cleaning properties, and in terms of environmental impact will consider easy to use, flexible, and adaptable [62]. 

### 2.1. The Role of Nanoparticles (0D Nanomaterials) in Developing Polymeric Membranes

Lately, nanocomposite polymeric membranes have captivated scientists’ attention towards applications, such as water purification, removal of microorganisms, chemicals, heavy metals in water and wastewater treatments. Using nanocomposite-based polymeric membranes for different technologies in wastewater treatments has developed limitations, so to enhance their performance, inorganic fillers must be added. The addition of different nanofillers/nanomaterials, for instance, carbon nanotube, zinc oxide, graphene oxide, silver, and copper nanoparticles, could overcome its limitations by amplifying hydrophilic properties, increasing retention of hazardous waste while enhancing thermal and mechanical stability [69,136,137,138,139].

Over the past decade, researchers developed artificial membranes for special operations with unique characteristics, for example, permeability, selectivity, specific chemical, and physical properties. To this extent, different methods accomplish, for example, track-etching, stretching, sintering, phase inversion, electrospinning, and interfacial polymerization [140]. For membranes, it has been used for preparation in diverse organic and inorganic materials. For instance, developing inorganic membranes from ceramic, glass, and metals. Organic membranes developed from composite materials or polymers [141]. Ceramic membranes achieve superior thermo-mechanical and chemical stability versus polymeric membranes. The hydrophilicity and the surface charge of ceramic membranes are superior to the polymeric membranes. As a result, ceramic membranes can endure higher pH and temperature in an excessive oxidizing environment, but polymers grant the flexibility and are cheaper [142]. Polymeric membranes must be performant, so must fulfill some requirements regarding the need to optimize and enhance the separation process [136], improving other physical properties, namely, stability, hydrophilicity, and fouling resistance [143].

Regardless of innovation in the membrane industry, there are multiple problems to overcome in order to proceed with large-scale implementations. One of the most important aspects of the membrane applications industry is membrane fouling [144], caused by conglomeration on membrane surfaces and pores. Therefore, contaminated membranes may absorb an enormous quantity of cleaning agents, damaging the surface, and rising costs of operation and maintenance of water treatment [145,146]. 

The longevity of the membrane from the industrial point of view is important but still needs to overcome some challenges regarding its complexity. Current developments in nanotechnologies have expanded the realm of membrane technology for the sake of refining water treatment. These polymers are exceptional, thanks to their dispersed nanoparticles in the polymeric matrix and perhaps, when used in contrasting processes, especially thanks to their unique properties, stability, and separation grade, having their use in water treatment processes [147,148].

Normally, nanocomposite polymeric membranes are created by adding nanoparticles as fillers into a macroscopic matrix, coating nanoparticles on the membrane exterior, or spreading the polymer solution before molding [149]. Considering these aspects introducing fillers into the polymer matrix provokes polymer-nanocomposite membranes considered (MMMs) [150] along with nano-enhanced membranes [151]. According to the above aspects, nanocomposite polymeric membranes are great alternatives to solve all these challenges. One of the most important applications of these membranes is in water treatment [152]. These types of nanoparticles-based polymeric membranes with nanoparticles dispersed into the polymer, form these nanocomposite membranes which can upgrade their performances such as permeability and selectivity in polymeric membranes [153]. 

Using nanocomposite polymeric membranes for water treatment is a challenging topic that will be pursued and researched, and all publications in this field stand as evidence of growing interest in solving the water problem. The following pages of the review will present the highest relevant fillers included in polymeric membranes. Table 1 presents the principal use of the nanocomposite polymeric membranes for water treatment. An analysis regarding the membrane technology and the use of the routine nanofillers used in polymeric membrane groundwork for water treatment is also displayed.

An extended overview of the fillers used in the formation of nanocomposite polymeric membranes for water treatment applications will be presented, highlighting the main advances and the influences of the presence of nanoparticles over their performances in environmental applications.

Titanium dioxide (TiO_2_) is an industrial pigment, disinfectant agent, and photocatalytic material [67,142,154]. TiO_2_ can be synthesized in three crystalline forms, noting that rutile and anatase are suitable for photocatalytic action [155]. TiO_2_ has excellent thermo-chemical stability, being less expensive than other nanomaterials, and also has low toxicity [152]. In addition to the photocatalytic properties, it has been intensively applied in water treatment processes and anti-fouling applications. The most important advantage of TiO_2_ nanoparticles is its interminable lifetime and the fact that it stays unaffected under degradable actions of organics and microorganisms. 

TiO_2_ nanomaterials present some other advantages besides the one mentioned above, which concern its hydrophilicity and oxidation capacity that transform it into a great material for applications in membrane fields. By developing new membranes with self-cleaning properties, the fouling rate can decrease but still maintain the water permeability of the membranes [69]. It relates to another issue regarding these nanocomposite polymeric membranes to the accumulation of the nanoparticles. TiO_2_ nanoparticles develop conglomerates which lead to efficiency decrement and to membrane performance. The mechanism of the TiO_2_ agglomeration has been intensively studied. Sotto et al. [156] examined the mechanism of nanoparticle agglomeration at low concentrations. The fabrication of PES-TiO_2_ nanocomposite polymeric membranes through adding ethanol influenced the TiO_2_ diffusion. Analyzing TEM images, some observe that adding ethanol stimulates the increase of cluster sizes. Through the agglomeration process, nanocomposite polymeric membranes enhanced permeability.

Instead, Abedini et al. [105] synthesized a new hybrid membrane based on TiO_2_ and cellulose acetate through the ex situ sonochemical methods. The water flux tests increased when nanoparticle concentration increased by 20wt%; thus, at 25wt%, TiO_2_ nanoparticles agglomerated on top of the membrane which conducted in the water flux decrement.

Vatanpour et al. [154] investigated some commercial TiO_2_ materials to test their performance on PES membranes, and they have shown that Degussa P25 exhibits better diffusion, which indicated enhanced hydrophilicity. This fact could indicate that the 20% rutile imprinted stability and enhanced antifouling properties.

Even if the membrane preparation is still in the beginning step and further studies must be done, researchers are trying to synthesize new membranes for diverse applications. Zhang et al. [157] investigated a novel approach by comparing two different methods of synthesis for TiO_2_ composite membranes through polymerization with polydopamine. Alam et al. [56] studied another interesting technique that involved the preparation of TiO_2_-based composite membranes. The TiO_2_/PES synthesized polymeric membranes prepared through the atomic layer deposition (ALD) technique, showed that the membranes exhibited up to 90% salts removal. Zhang et al. [158] mentioned in their work, the nanocomposite fibers based on PES and TiO_2_ prepared through the electrospinning method. Results conducted showed better porosity and hydrophilicity of the composed membranes, and the flux rate increased by 19%.

Silver salts and nanoparticles, also in polymers and metal oxide composites, being some of the most frequent examples used in environmental protection and biomedical applications, mainly because of their antibacterial properties. Zapata et al. [159] reported the use of silver nanoparticles in many applications, such as health and environmental protection. Mainly thanks to the possibility of incorporation into polymers, this could bring great potential in nanofibers membranes. The World Health Organization (WHO) reports that it could use Ag salts in drinking waters as a bacteriostatic agent; though, WHO also stated the daily intake of Ag should not be higher than 0.1 mg/L to be tolerated [160].

Chaloupka et al. [161] has also reported that the antibacterial activity of Ag nanoparticles depends on the size, shape, and chemistry of the nanoparticles. Because of the synergism between the nanoparticles and the release mechanism, Ag nanoparticles could decrease the bacteria spread which could negatively influence the permeation and respiration of the membranes [69,162].

There are multiple casting solutions for Ag nanoparticles incorporation on the membrane surface, such as direct adsorption reduction or in situ synthesis [163]. Cao et al. [164] synthesized a new membrane by incorporating Ag nanoparticles into the membrane’s surface through the vitamin C as a reducing agent. Zhu et al. [165] reported an experiment on the evaluation of membrane’s performances by immobilizing metallic silver on chitosan-based polymeric membranes. They also evaluated the antibacterial effect with the help of some bacteria responsible for developing biofouling problems. Some observed that the incorporation of chitosan into membranes does not influence the bacteria development, but adding silver brought great antibacterial effects.

Haider et al. [166] evaluated the antibacterial activity against *E. coli* of the PES membranes by incorporating Ag nanoparticles into the membranes. After testing the polymeric membranes, they have shown that the amino groups found on the surface of the membranes could reduce the colony spread and enhance the disinfection potential. 

Similarly, Biswas and Bandyopadhyaya [167] prepared PES membranes by surface modification using Ag nanoparticles by generating -SO_3_H groups on the membrane surface. Andrade et al. [168] prepared Ag nanoparticles in situ, having nano cubic and spherical shapes dispersed equally on the membrane surface. It is worth mentioning that adding the stabilizing agent AgNO_3_ enlarged the dimensions of the nanoparticles, and when decreasing the sizes of the nanoparticles, they could enhance the antibacterial activity of the membranes. Rehan et al. [82] presented an ex situ method for the synthesis of Ag nanoparticles by using AgNO_3_ and PVP as capping agents that produced an enhanced antibacterial effect. 

Escobar et al. [86] and Andrade et al. [87] reported the preparation of nanocomposite membranes through an inverse process using stabilized silver nanoparticles, and both concluded in having better permeability. Usually, researchers use commercially available silver nanoparticles, but some studies used bio-silver nanoparticles synthesized with *Lactobacillus fermentum* [169,170]. In conclusion, they found that the incorporating the bio-silver nanoparticles in the membrane composition increased the water flux rate and stability.

Copper (Cu) and its derivatives present antibacterial and antifungal effects upon different microorganisms [129,130,171,172]. There are still some uncleared issues regarding the interaction of Cu nanoparticles with some bacteria which generated ROS and other oxides anions [173]. Cu^2+^ ions may link with thiol groups from the molecules of DNA and proteins and can disrupt the biological and chemical processes that could lead to protein denaturation [169,170]. Like silver, copper presents antibacterial property, low cost and sorption properties [174,175,176] also possesses applications in the biomedical and textile industry [177], packaging materials, and also water treatment and can control biofouling production [178,179,180].

Cu nanoparticles have been extensively used in applications for nanocomposite polymeric membranes. Xu et al. [181] and Xu et al. [182] have reported antibacterial copper (II)-chelated PAN membranes. Some researchers compared two membranes, one based on Cu-PEI (polyethyleneimine) which showed lower hydrophilicity; however, the membrane-based on PAN-PEI-Cu proved high water permeability but still low hydrophilicity. Considering the presence of Cu^2+^, the membrane presented a 95% antibacterial effect, and the biofilm formation delays. After evaluating the Cu-NPs-PES membranes, Akar et al. [98] presented that water properties decreased as the polymer concentration increased. To promote better anti-fouling performance in activated sludge as a fouling solution. 

Zinc oxide (ZnO), one important inorganic nanomaterial with important applications because of its antibacterial and physical properties. Because of their functionality, ZnO nanoparticles attract hydroxylic systems [183], simultaneously, their exterior seems to be larger than other inorganic nanomaterials [184]. Introducing this inorganic filler resulted in the improvement of the polymer characteristics [185,186]. The addition of ZnO nanoparticles into the membranes created better adsorptive capacity for the membranes based on ZnO filler. These fillers enhanced also the anti-polluting endurance throughout the separation of diverse pollutants existing in waters [187]. 

Moreover, it proves that adding ZnO into the membranes enhanced their fouling performance and also prolong the durability of the membranes [188]. For instance, adding ZnO nanoparticles into membranes has a significant advantage as an efficient elimination of heavy metals from wastewaters. They attribute the adsorbent property of the ZnO materials to their electropositive nature. Finally, it can state that incorporating ZnO nanomaterials in different membranes for wastewater treatment has satisfactory results and showing good adsorbent performances [189].

Using materials such as aluminum oxide (Al_2_O_3_) nanoparticles for synthesizing composite polymeric membranes showed an improvement of the hydrophilicity and fouling property in the obtained polymeric membranes [190,191,192,193,194]. The most common methods used in the synthesis of the membrane are interfacial polymerization [190], surface deposition and structure entrapment [191], and also in situ polymerization [192]. Fe_2_O_3_ and Fe_3_O_4_ possess many spectacular characteristics which include magnetic properties, antibacterial, antifungal, and hydrophilic properties. Zinadini et al. [129] revealed significant results of Fe_3_O_4_ nanoparticles on the formation of PES membranes for applications in dye removal.

Niksefat et al. [195] developed nanocomposite membranes for FO applications by studying the morphology and performance effects of silica nanoparticles. These types of membranes confirmed water permeability and flux increment with increasing silica content. Kebria et al. [196] investigated another model of SiO_2_-PEI composite membranes for NF applications such as dye removal from organic solutions. 

### 2.2. The Role of 1D Nanomaterials in Developing Polymeric Membranes

This section of the review provides a brief overview of the use of one-dimensional nanomaterials such as nanotubes and nanofibers/wires in developing polymeric membranes for water purification. In recent years, 1D nanostructures have gained great interest because of their unique properties and interesting applications. They pose promising features for using them, particularly in the design of porous nanostructures with beneficial use for catalysis, bioengineering, environmental protection, sensors, and also photovoltaic devices because of their high surface area [197,198]. The next section of the manuscript will provide some examples of such nanomaterials.

Carbon nanotubes (CNTs) gained scientists attraction thanks to their extraordinary properties and antibacterial effect. CNTs enhance the potential of membranes for other applications but alter the physicochemical properties of membranes. Normally, by increasing the mechanical and thermal properties of the membranes, CNTs nanopores enhanced permeability without decreasing selectivity [199]. Adding CNTs into PES has shown polymeric membranes to increase water flow and decrease the fouling rate, thus increasing the hydrophilicity of PES membranes, demonstrated by Celik et al. [59]. The uncovered membrane has low permeability compared to the membrane covered with a thin layer of CNTs [112]. Similarly, the nanocomposite membrane based on nitrocellulose coated with a film containing 3% CNTs, showed significant antimicrobial activity of 80–90% against Gram-positive and Gram-negative bacteria [113]. As a result, CNTs have functions in wastewater treatment since they did not show toxicity against fibroblast cells.

Shah and Murthy [116] have prepared composite polymeric membrane functionalized with CNTs-polysulfone to remove heavy metals. These types of composite polymeric membranes have a high percentage in removal of heavy metals by increasing the loading of MWNTs. In addition, Shen et al. [117] have synthesized nanocomposite membranes embedded with methyl methacrylate (hydrophobic) modified multi-walled CNTs by interfacial polymerization. This type of membrane has 99% rejection for Na_2_SO_4,_ and the water permeates flux was enhanced by 62% compared to the pristine thin-film composite membranes. 

Adding different fillers lead to increased permeability and decreased fouling tendency [118]. Normally, most syntheses of CNTs use acids before incorporating them into the membranes, but the treatment of the membrane with acid could disrupt the CNTs. To solve this problem, Sianipar et al. [119] suggested covering the CNTs with polydopamine to avoid wall disruption, which resulted in proving 99% rejection rate, and the water permeability increased by 19–20% and also having better stability of the membrane.

Lately, carbon nanotubes have attracted great interest in applications for wastewater treatments. Thus, membrane bioreactors incline to have fouling issues, Khalid et al. [120] fabricated a new composite membrane based on PEG-CNTs through the processes of membrane bioreactors and it proved that adding CNTs into the membrane composition increased fouling and performance.

Another important type of nanomaterial used for application in the removal of dye pollutants from water were nanofiber adsorbents. This type of nanofiber adsorbents is usually made through the electrospinning process and is superior to nanoparticle-based adsorbents, mainly because of their easy recovery. A good example of nanofibrous adsorbent material is polyethersulfone (PES) electrospun nanofibers with di-vanadium pentoxide (V_2_O_5_) nanoparticles, which has been effectively used for the removal of MB dyes from pollutant water [200]. Electrospun nanofibrous membranes have been widely used for ultrafiltration and nanofiltration. These types of membranes were developed for their size exclusion of contaminants having large porosity and interconnected structure with micropores which enhances fouling resistance. For instance, it has been developed a PES electrospun nanofiber mat coated on a poly(ethylene terephthalate) (PET), in order to evaluate it as a membrane for liquid filtration and removal of polystyrene (PS) particles from water [201]. Therefore, to exhibit a high potential for water treatment, it should be mechanically reinforced and hydrophilized to raise water permeability and flux. To fulfill these aspects, PES nanofibers were stabilized and hydrophilized by incorporating ZrO_2_ [202] and TiO_2_ [203] nanoparticles. Another issue that nanofibers must overcome is the biofouling challenge, which could be done through the sake of antibacterial nanoparticles such as Ag nanoparticles incorporated within the nanofibers. For FO (forward osmosis) water treatment, Pan et al. [204] studied a TFC (thin film composite) membrane for the removal of diverse pollutants. The results proved that the as-formed membrane offers remarkable bactericidal effect for *E. coli* and *S. aureus*.

### 2.3. The Role of 2D Nanomaterials in Developing Polymeric Membranes

Researchers and scientists are developing new two-dimensional materials for the synthesis of nanocomposites with applications in water treatment [205]. Next, will present some 2D materials used for the design of water treatment membranes such as graphene and graphene oxide but also some clays, which are just some of them. The 2D composites are so interesting and have captivated researchers due to their unique properties such as chemically active surface area, which can enhance significantly the transportation features regarding their shape, size, and structure [206]. 

Worth mentioning that the transportation features of such 2D materials could bring new outcomes and challenges to the synthesis of nanoporous composite with applications in desalination membranes [206,207]. Graphene was the first 2D material used in water treatment, which was studied due to its special characteristics based on the honeycomb arrangement structure, which presents good permeability in developing such membranes. Moreover, graphene was developed mainly for the applicability of two important usages: water desalination [208] and water purification [209]. Graphene has exhibited good practicability in reverse osmosis used especially for water desalination, the material being able to remove salts up to 100% [208]. In the case of water purification, graphene coated on PVDF exhibited rejections >99% for organic dyes. 

Graphene oxide (GO), carbon nanomaterial resulted from graphite treated with strong oxidizers, exhibiting balanced hydrophilic character according to the oxidation level, different from graphene, which is hydrophobic [188]. They have shown that graphene oxide enhances the thermo-mechanical effects of polymeric membranes [210]. The GO presents functional groups on the surface that can transform into hydrophilic functional groups (-NH_2_, -OH, and -SO_3_H), which are useful in producing functionalized graphene oxide and graphene-based materials [211,212]. GO gained important consideration in applications regarding nanocomposite polymeric membranes for water treatment, inclusive in water purification, also examined as potential candidate for the removal of pharmaceuticals and organic molecules from water and wastewaters [212,213]. Like other nanofillers already presented in this paper, GO incorporation in polymeric membranes can bring significant change to the hydrophilic profile and improving also permeability performance [123]. 

Yang et al. [214] reported studies regarding the improved flux and anti-fouling capacity of nanofiltration membrane by incorporating GO and enhanced dye retention up to 90%. Another example of membranes used in dye molecule removal was GO impregnated with TiO_2_ composite films having application in water purification [215]. As another option, developing GO membranes functionalized with different nanomaterials could enhance their performance, which could lead to the heavy metals and salts elimination from wastewaters [216]. 

In a similar case, new membranes for water desalination through the development of GO-based composite membranes have been studied [217]. For instance, Yin et al. [218] improved the removal rate for different salts in the case of GO nanocomposite membranes for water purification. In similar experiments, Chae et al. [131] have reported an almost 99% removal for NaCl in the case of preparation the GO-PA membranes. Adding GO into the membrane structure enhanced the water permeability and anti-biofouling capacity of these membranes [94]. Ali et al. [219] and He et al. [132] have reported a 97% rejection for salts using also membranes based on PSF integrated with GO.

Recently, graphene’s performance has been studied for wastewater treatment also. Yang et al. [220] synthesized composite membranes based-graphene to remove phthalates and pharmaceuticals from water media. In their results, authors observed that the rejection rate for phthalates was 99% and for pharmaceuticals between 32 and 97%. Crock et al. [134] developed polymer-composite membranes based on graphene feathered with Au nanoparticles and then included into PSF. These types of membranes exhibited remarkable removal of up to 69% for compounds such as dextran, showing that this membrane based on graphene has important relevancy in water treatment. 

Xiaowei et al. [221] have prepared chitosan/GO chitosan-derived membrane for water desalination. The authors carefully examined the morphological transformations, wettability, and also desalination ability of the membranes by changing the GO amount. The results showed an improvement in the permeate flux (30 kg/m^2^h) and salt rejection by 99.99% at 81 °C observed for NaCl (5 wt.% aqueous) solution with 1 wt.% GO concentration. In conclusion, the stability of the chitosan/GO membrane enhances because of the good compatibility between GO and chitosan biopolymer. Another example of GO with chitosan-derived materials for water desalination is presented next. Deng et al. [222] reported in their work that chitosan/GO/titania oxide membrane show about a 30% higher salt rejection rate than the GO membrane.

Another 2D material used for water treatment was di-vanadium pentoxide (V_2_O_5_) used for the preparation of water treatment membranes. Gao et al. [209] synthesized a new multifunctional membrane based on hierarchical TiO_2_/V_2_O_5_ with applications in water purification, which exhibited a high removal rate of up to 90% for total organic carbon (TOC), high quality in water filtration, and also reduced membrane fouling. This membrane used in photodegradation techniques for organic pollutants showed that the membrane has great potential for water purification.

### 2.4. The Role of 3D Nanomaterials in Developing Polymeric Membranes

Currently, there are more inorganic materials with a 3D structure, such as zeolites and silicate, used for water treatment applications. The usual applications for zeolite membranes were in desalinization, gas, and liquid separation, and also in membrane reactors [223,224,225,226], showing unique properties such as a better permeability and selectivity, but also great thermal and mechanical properties, which presented interest for using in diverse applications [227]. Adding zeolites into the composition of these membranes was beneficial regarding the enhancement of the hydrophilicity and molecular character of the developed membrane. Different authors reported positive results regarding the permeability and stability of the zeolites on the RO membranes, which was described by Huang et al. [228]. Another study by Dong et al. [229] presented the progress of using PS supports functionalized with zeolite NPs in the thin-film nanocomposite NF membranes fabrication. These membranes exhibited enhanced permeability and salt removal of 93.4%.

The authors presented a study regarding carbon dioxide adsorption or chemical fixation through the help of a mesoporous zeolite/chitosan composite membranes. After submitted to different tests, the zeolite/chitosan composite membrane showed great results on the adsorption of CO_2_ compared to raw chitosan or zeolite. Moreover, the nanocomposite membrane exhibited excellent catalytic activity in the chemical fixation of CO_2_ [230].

Silicon dioxide or silica (SiO_2_) is an important inorganic metal oxide used as filler for developing composite membranes for water purification. Because of its interesting features, it has been intensively used in diverse applications such as accessible manipulation, low reactivity, and also for its good chemical properties [69]. For example, Ahmad et al. [231] synthesized PS membranes by incorporating a diverse amount of silica at different concentrations. That silica is hydrophilic, permitted the increment of permeability up to 17.32 L/m^2^h in comparison with the simple membrane which was at 1.08 L/m^2^h. The results suggested an enhancement in antifouling properties, which indicated a better oil-in-water separation. 

## 3. Chitosan-Based Nanocomposite Polymeric Membranes: A Case Study

In recent decades, water scarcity has become an increasing problem that needs to be solved. We know that water is a basic human need for our survival, and also is the most valuable and permanent resource in the world. However, this aspect is changing, mainly, because of our rapid industrialization where human society is contributing to diminishing the water resources [232,233,234,235]. From these aspects, water scarcity has developed into an arising problem, and the need to engineer new materials to resolve it has become imperative. The main interest was focusing on finding novel synthesis methods to develop materials with applications in water treatment. To sustain diverse activities in various industries or other processes, water sources must be purified. The water purification process implies removing chemical contaminants from polluted aqueous systems [153,236].

In the last years, new and promising polymeric membranes for water treatment were developed [236,237]. Various membranes made from polymer materials coated with different biomaterials exist which have immense applicability in decontamination and water treatment (Table 2). Membranes have the purpose of removing fine and large particles; however, the process depends very much on the pore size of the membrane [238].

Different processes such as microfiltration, nanofiltration, ultrafiltration, and reverse osmosis use diverse particles. There are other processes such as conventional filtration that filter only small particles that could not see with the naked eye, whereas microfiltration isolates only particles larger than 10 µm, such as microorganisms, for instance. The ultrafiltration method is used for the separation of smaller particles than 1000 Å, such as macromolecules or colloids. Nanofiltration and reverse osmosis membranes usually can separate ions and salts, but both need pressure to work [259,260,261,262]. 

After analyzing and comparing multiple types of membranes, biopolymeric membranes have attracted great interest since they are cost-efficient, biodegradable, safe for humans, animals, and the environment. After examining the challenges in obtaining pure drinking water and the above characteristics, they have proven that the most used material is chitosan. It has been shown that the use of chitosan-like biopolymers and their impregnation with various biomaterials could improve the versatility of membranes and their effectiveness [237,263]. As already mentioned in the previous section of the review, one must consider the most important factors affecting the adsorption performance of the chitosan membranes. It is essential to establish the critical point of view regarding the use of chitosan membranes for water purifications. However, the physical characteristics of the material, source and composition, physical properties, and process application are the most significant aspects which influence the adsorption processes of the membranes [264]. The chitosan physical character has the most important effect on the adsorption capacity of the chitosan-based membranes. To be used for water treatment, chitosan has been physically changed into different forms such as hydrogels and beads [265,266], nanoparticles [267], fibers [268], powder [269], flakes [270], membranes [271], and honeycomb or sponge-like structures [272,273]. The second most important aspect that must take into consideration for establishing the adsorption capacity of the polymeric membrane is the source and composition of the chitosan. As known, chitosan is extracted from crab shells and lobsters, and other natural materials that have been used for water purification. The adsorption capacity of the chitosan membranes depends on the binding ability of metal ions with the amino groups of the chitosan, degree of deacetylation, and also density of amino groups on the surface [13]. Another important factor for the adsorption capacity of chitosan membranes are its physical properties such as surface area, volume of the pore, density, and elemental composition [274]. It proves, also, that process factors such as temperature, pH, presence of anion or cation, and degree of combination are very important in the chitosan processing. For instance, increasing the temperature during the process could cause an increment in the adsorption rate by lowering the adsorption capacity [275].

In recent years, researchers have shown that adsorbent membranes can be used in environmental applications such as water purification, and potentially be used to separate or remove dangerous compounds, such as heavy metals and other toxic compounds presented in wastewaters. To detect contaminants and pollutants from water sources, nanocomposite polymeric membranes-based on chitosan were investigated. Chitosan-based biopolymers are very important because of their high content of amino and hydroxyl groups, good biocompatibility, biodegradability, nontoxicity, reactivity, good hydrophilicity, and cost-effectiveness, which make them essential in water treatment applications [188,261,262,276]. Chitosan presents hydroxyl and amine groups on its surface, the reason for their wide use in heavy metal removal from wastewaters [276,277,278,279,280]; however, it does have some inconvenience when it comes to its low stability, thermo-mechanical properties and, also, porosity [281]. To overcome these difficulties related to issues, scientists developed chitosan-based adsorbent membrane through diverse methods to defeat these drawbacks [237,282,283,284,285]. This chapter of the review is focused on offering a perspective regarding the preparation and applications of chitosan-based polymeric membranes with application in wastewater treatment [286].

Various membranes based on chitosan have been developed for using them in multiple applications, but the most special use was to remove toxic pollutants from contaminated waters. 

Singh et al. [286] prepared chitosan-based thiomer using the microwave irradiation method to remove arsenic from contaminated waters. The chitosan thiomer shows high efficiency in removing the pollutant from the groundwater, showing an 85.4% elimination of As(III) and 87% for As(V). Worth mentioning that the synthesized thiomer is efficient even at 10 ppb and can reduce the arsenic concentration to less than 3.2 ppb, which is much lower than the WHO guidelines.

Tang et al. [287] made chitosan (CTS) with polyvinylidene fluoride (PVDF) in a mixture (CTS/PVDF) for synthesizing a composite using a single-step process. The composite polymeric membrane synthesized based on chitosan has excellent mechanical strength, better adsorption capacity, and good selectivity compared to Cd(II) over Pb(II). The tests suggested that the adsorption capacity was in a normal range. 

Gedam et al. [288] prepared a new type of chitosan-based on iodate used for removing the lead ions from wastewaters. Using the Langmuir and Freundlich models could measure the adsorption capacity for Pb(II) ions. Lei et al. [289] studied a mineral based on apatite and chitosan, showing that hydroxyapatite has great stability, low water solubility, and high retention capacity. The adsorption capacity for this nano-hydroxyapatite is ranging from 208.0 to 548.9 mg/g. In conclusion, this nano-hydroxyapatite chitosan composite adsorbent confirms its efficiency in removing Pb(II) from wastewaters. 

Wang et al. [290] used immersion precipitation for the adsorption of Cu(II) on a porous chitosan membrane. To develop a chitosan membrane that has a porous structure, silica has been introduced as porogen material. The as-obtained polymeric membranes presented a reticular structure having pores ranging from 1.9 to 4.6 µm and an adsorption capacity of 87.5 mg/g.

Razzaz et al. [291] fabricated through two techniques, chitosan/TiO_2_ composite nanofibrous adsorbents. After evaluating these type of nanofibers, they showed that are efficient for removing Cu(II) and Pb(II) ions. They demonstrated that chitosan/TiO_2_ composite adsorbent presented high adsorption capacity than TiO_2_-coated chitosan nanofibers. 

Habiba et al. [292] prepared an interesting type of membrane-based on chitosan/polyvinyl alcohol (PVA)/zeolite nanofibrous composite through the electrospinning method. Chitosan/polyvinyl alcohol/zeolite membrane has good efficiency in removing toxins from wastewaters. It also determines that the synthesized composite polymeric membrane presented regeneration and reusability capacity and no loss of adsorption capacity. Zavareh et al. [293] fabricated Cu-chitosan/Fe_3_O_4_ nanocomposite with applicability in phosphate removal from wastewaters. This nanocomposite presented a high rate of adsorption capacity of 88 mg/g^−1^ than raw chitosan. Zuo et al. [294] synthesized an innovative adsorbent based on poly(vinyl alcohol)/citric acid/chitosan (PVA/CA/CHT, PCC) for the removal of Cr(III) from aqueous solutions.

Mohseni-Bandpi et al. [295] developed a new adsorbent based on magnetite-chitosan composite used in applications for the removal of fluoride from wastewaters. With this type of membrane, chitosan loaded with magnetite nanoparticles and noticed that pH, concentration, and temperature enhance the adsorption capacity of the adsorbent. They observed that the adsorption capacity influences the changing of the pH, temperature, volume, and even the concentration of fluoride solution.

An interesting functionalized chitosan-based membrane was developed by Makaremi et al. [296]. To enhance the filtration capability, mechanicals, and antibacterial activity of the chitosan membrane, they have functionalized it with nanofibers of polyacrylonitrile and ZnO nanoparticles. After testing this type of porous membrane, it proves that adding the nanofiber and nanoparticles enhanced the adsorption performance for Cr(III). Another material such as chitosan-based tetraethyl orthosilicate/aminopropyl triethoxy silane composite membrane was efficient in the removal of heavy metals from wastewaters [297]. 

Chitosan and chitosan-based systems have uncovered a great potential in the detoxification of pollutants from waters and have shown great performance in the removal of toxic metals, such as Cr(VI) [298,299], As(V) [286,300,301], Mo(V) [302], Hg(II) [303], Cu(II) [283,296], and Pb(II) [304,305], but also have presented potential in removing inorganic species such as nutrients (NO_3_^−^, NO_2_^−^ and PO_4_^3−^) [306,307]. In addition to the diminishing of inorganic pollutants from wastewaters, chitosan was used as an adsorbent for organic pollutants [39].

As already presented in the previous section of the paper, chitosan-based materials have been shown to have perspectives as adsorbents in wastewater treatment, such materials are metals [301], metal oxides [304], and bimetals [305]. Another important application of chitosan-based ZnO nanoparticles is in agriculture for the removal of permethrin [308]. Some researchers tested the adsorbent in agriculture, and showed that permethrin’s removal capacity increased from 49 to 99% when it uses the chitosan-based ZnO nanoparticles [309]. 

Saifuddin et al. [310] tested also a variation of chitosan impregnated with silver nanoparticles (Cs-Ag NPs) used also in agriculture for the removal of pesticide atrazine (a persistent herbicide). While testing this composite, researchers discovered that the pesticide content decreased when increasing the adsorbent dose. Danalioğlu et al. [311], in their study, reported information about using some composite systems based on magnetic-activated carbon/chitosan used to annihilate diverse antibiotics (ciprofloxacin, erythromycin, and amoxicillin). When testing the composite system for antibiotics, they noticed a good adsorption performance. To the example above, Nadavala et al. [312] developed a system based on chitosan-sodium alginate-calcium chloride used for the removal of phenols. The results sustained that the as-obtained system exhibited the optimal adsorption capacity for phenols. 

In addition to the inorganic pollutants, organic pollutants such as dyes, phenols, pesticides, detergents, play an important role that strongly influences the quality of the waters and their removal will be displayed next. Every year, about 9 million barrels of oil are being discharged into the ocean worldwide. Researchers are trying to develop new environmentally and supportive approaches to remove oil pollutants from waters without threatening the ecosystems [39]. Ummadisingu et al. [313] studied and tested chitosan extracted from seafood industry wastes and purified it for further applications in the removal of oil from diverse polluted waters. In conclusion, researchers stated that pH, concentration, and contact time influence the adsorbent quality of chitosan [313]. The study conducted by Bibi et al. [314] described the adsorption capacity of a membrane-based chitosan, nanotubes, and PVA with silane for the removal of naphthalene [314].

Fan et al. [315] developed a novel magnetic chitosan/graphene oxide (MCGO) adsorbent for the removal of methylene blue. To determine the structural and morphological modifications and magnetic properties of the MCGO adsorbent, XRD, SEM, and FTIR techniques were used. The authors changed some parameters to establish the adsorption rate. The results indicated that the adsorption of methylene blue into the MCGO (95.16 mg/g) was higher than chitosan (60.4 mg/g). Nitrophenol, another organic pollutant that needs to be eliminated from water by using chitosan-based nanocomposite polymeric membranes. Khan et al. [316] synthesized chitosan/GO nanocomposite coated with Cu nanoparticles to identify and eliminate 4-nitrophenol. The results showed a constant reduction rate, good sensitivity, and good detection.

Herman et al. [317] studied the synthesis and characterization of chitosan-silica membranes for treating hotel wastewaters as being affected by polyethylene glycol and polyvinyl alcohol. The purpose of the study was to determine the mechanical features, permeability, selectivity, and also the effect of membrane’s composition in order to see the change in their performance. Experiments showed that adding higher amount of PEG enlarged the pore diameter but increases the flux rate; although, the rejection value was smaller. In contrast, adding PVA shrank the pore diameter and increased the rejection value but decreased the flux rate. Another study made by Rosdi et al. [318] investigated the purpose of adding silica in improving chitosan membrane performance in removing Pb(II) ions from water solutions. The results indicated that the composite polymeric membranes presented greater efficiency for lead removal compared to pure chitosan membranes.

Another example implies chitosan/zeolite composite membranes for the elimination of trace metals ions such as Cr, As, Cd, and Pb from wastewaters. For this study, researchers used chitosan combined with zeolites for developing composite membranes filled with glutaraldehyde to eliminate as mentioned metal cations through the evacuation permeation process (EPP). In conclusion, results indicated the potential capacity of using the chitosan/zeolite composite membranes in wastewater purification [319,320].

Carbon nanotubes were considered valuable materials in combining them with chitosan for developing adsorbent membranes for heavy metals removal [321,322]. Special attention is brought to the chitosan/biochar (BS) composite which was used to give higher performing adsorbent capacity to the obtained polymeric membrane. Due to the porous structure of biochar, it has been studied in adsorbent membranes with important applications, such as removal of heavy metals, phosphates, and various antibiotics and pharmaceuticals from waters [321,322].

## 4. Conclusions

The manuscript highlights the preparation and applications of composite membranes with special attention on chitosan-based membranes for the removal of pollutants from aqueous systems. Chitosan, the most used biodegradable, biocompatible, hydrophilic biopolymer, presents considerable features for using it in applications such as biomedicine, biosensors, food industry, cosmetics, agriculture, but its most evaluated usability remains water treatment. This review presents a summary regarding diverse materials used in developing innovative chitosan-based nanocomposite membranes for water purification. 

Analyzing the presented nanocomposite polymeric membranes in the manuscript, it is worth mentioning some suitable examples that could bring important benefits to the water treatment industry. The excellent antimicrobial activity of chitosan has gained great interest by using it in membranes for industrial desalination. Chitosan-functionalized with graphene oxide offers an excellent adsorbent for the heavy metal removal from wastewater systems. Moreover, introducing an inorganic filler, ZnO in chitosan’s membrane composition has demonstrated improvements in the adsorptive capacity, fouling performance, and also durability of the membranes. In addition to the examples above, an innovative magnetic chitosan/graphene oxide (MCGO) adsorbent for the removal of methylene blue from wastewaters has been developed, but additional examples should be highlighted. 

The synergism between chitosan and the reinforcing agents was also debated within the manuscript. It was found that using this type of fillers in the composition of the chitosan-based membranes brings significant improvements in their performances. Moreover, the addition of various nanomaterials as fillers (C nanotubes, ZnO, Ag, Cu, and TiO_2_) could overcome their limitations by increasing retention, hydrophilicity, and thermo-mechanical stability, and also swelling, which prevents its use at a larger scale. Thus, this significant accomplishment would be a great breakthrough for the water treatment industry. Broadening chitosan’s applicability in nanocomposite membranes, could overcome its limitations and bring major outcomes. This review presents a perspective on the promising potential of chitosan-based nanocomposite membranes in developing innovative materials for water treatment applications.

## Figures and Tables

**Table 1:** Applications of Nanoparticles in Nanocomposite Polymeric Membranes for Water Treatment.

Nanoparticle	Membrane Type	Targeted Application	Ref.
ZnO	MF	Wastewater Treatments	[63]
		Copper ions elimination	[64]
		VOC elimination from water systems	[65]
	UF	Humic Acid (HA) elimination	[66]
		Salt elimination	[67]
		Micelles elimination from solutions	[68]
		Pollutants removal (alginate and HA)	[69]
		Evaluating antifouling properties in diverse water membranes, model-Bovine serum albumin (BSA)	[70]
		Treatment of wastewaters	[71]
		Bacterial removal from aqueous solutions	[72]
	NF	Water treatment (HA elimination)	[73]
		Salt and metal ions elimination	[74]
		Separation of Rhodamine B	[75]
		Inorganic salts and HA elimination	[76]
	FO	Salts tests elimination (model of MgSO_4_)	[77]
		Desalinization and wastewater treatments	[78]
	RO	Salt, bivalent ions, and bacterial retention models	[79]
AgNO_3_	UF	Evaluating antifouling properties in water membranes—Mixture model—BSA.	[80]
Ag-NPs	MF/UF	Evaluating antifouling properties in water membranes-Mixture model—BSA.	[81]
	UF	Water purification	[82]
		Evaluating antifouling antibacterial properties in water membranes—Model bacteria—*E. coli*	[83]
		Evaluating antifouling antibacterial properties in water membranes—Model bacteria—*E. coli.* Mixture model: BSA and dextran	[84]
		Evaluating antifouling antibacterial properties in water membranes—Model bacteria—*P. putida*. Mixture model: BSA	[85]
		Evaluating antifouling properties in water treatment membranes	[86]
		Test mixture: polyethylene glycol (PEG) and dextran solutions	[86]
	NF	Evaluating antibacterial properties in water treatment membranes: *E. coli*, *S. aureus*	[87]
AgNO_3_	NF	Evaluation of antibacterial properties and salts elimination (Na_2_SO_4_). Model bacteria: *E. coli*	[88]
Ag-NO_3_	RO	Evaluating antibacterial properties and salts removal (NaCl). Model bacteria: *E. coli*, *P. Aeruginosa*, *S. aureus.*	[89]
		Evaluating antibacterial properties and salts (NaCl). Model bacteria: *E. coli*, *Bacillus subtilis*	[90]
		Evaluating antibacterial properties. Model bacteria: *E. coli*, *Bacillus subtilis*	[91]
	DCMD	Desalination of seawater through silver nanoparticles deposition	[92]
	PRO/RO	Evaluating antifouling and antibacterial properties in water treatment membranes. Model bacteria: *E. coli*	[93]
Ag-NPs	PRO	Evaluating antifouling and antibacterial properties in water treatment membranes. Model bacteria: *E. coli*, *Bacillus subtilis*. Mixture model: *C. testosterone*	[94]
bio-Ag_0_	UF	Evaluating antifouling and antibacterial properties in water treatment membranes. Model bacteria: *E. coli*, *P. aeruginosa*	[95]
	NF	Evaluating antibacterial properties and salts removal (Na_2_SO_4_). Model bacteria: *E. coli*, *P. aeruginosa*	[96]
		Evaluating antibacterial properties and salts removal (Na_2_SO_4_). Model bacteria: *P. aeruginosa*	[97]
Cu-NPs	UF	Evaluating antifouling and antibacterial properties in water treatment membranes. Model bacteria: *P. putida*. Mixture model: BSA	[85]
		Wastewaters treatment and evaluating antifouling properties in membranes. Mixture model: BSA	[98]
	RO	Evaluating antibacterial properties in water treatment membranes and salts removal (NaCl). Model bacteria: *E. coli*, *P. aeruginosa*, *S. aureus.*	[99]
TiO_2_-NPs	MF	Evaluating antifouling properties using whey solution	[100]
	UF	Evaluating antifouling properties in water treatment membranes. Mixture model: HA	[101]
		Evaluating antifouling properties in water treatment membranes. Mixture model: BSA, PEG, and MgSO_4_	[102]
		Treatment of water systems	[51]
		Evaluating UV-cleaning and antifouling properties. Mixture model: BSA	[103]
		Evaluating antifouling properties and salts rejection (NaCl). Mixture model: BSA and pepsin.	[104]
		Filtration systems	[105]
		Evaluating UV-cleaning properties and antifouling properties. Mixture model: red dye and BSA.	[106]
TIP		Evaluating antifouling properties. Mixture model: BSA	[107]
TiO_2_-NPs	FO	Evaluating salts rejection (NaCl)	[108]
	MF/MBR	Evaluating antifouling properties. Mixture model: BSA, PEG, MgSO_4_	[109]
nano-TiO_2_	MBR	Evaluating membranes based on algae	[110]
TiO_2_-NPs	NF	Filtration systems applications	[111]
CNTs	NF	Evaluating antifouling and salts rejection (NaCl, Na_2_SO_4_).	[112]
	NF	System purification	[113]
	UF	Water system treatments and biofouling application	[59]
	NF	Filtration systems applications	[114]
	NF	Filtration systems	[115]
	NF	Metal rejection (Cr(IV), Cd(II))	[116]
	NF	Treatment for salts elimination (NaCl, Na_2_SO_4_)	[117]
	NF	Evaluating antifouling properties in water treatment membranes	[118]
	UF	Water treatment for UF applications	[119]
	UF	Wastewaters treatment through a membrane bioreactor	[120]
	MF	Effluent treatment through a membrane bioreactor	[121]
GO	MF	Effluents elimination with high dyes content	[69]
		Wastewaters filtration	[122]
		Natural organics removal	[123]
		Water systems treatment	[124]
		Elimination of organic pollutants in salty water	[125]
		Distillery effluent treatments	[126]
GO	NF	Na_2_SO_4_ removal	[127]
		Soft water production	[128]
		Evaluation of dyes removal	[129]
		Water treatments	[130]
	RO	Salt removal (NaCl)	[131]
		Salts removal (NaCl, CaCl_2_ and Na_2_SO_4_)	[132]
	FO	Desalination processes for seawater	[133]
Graphene	UF	Wastewater treatment	[134]
	NF	Water purification	[135]

UF—Ultrafiltration, MF—Microfiltration, NF—Nanofiltration, FO—Forward Osmosis, RO—Reverse Osmosis, DCMD—Direct Contact Membrane Distillation, MBR—Membrane Bioreactor; titanium tetra-isopropoxide—TIP.

**Table 2:** Diverse Applications of Chitosan with Various Compounds.

Material Type	Compound	Selected Applications	Ref.
0D	AgNP_s_	Wound dressing applications	[32]
		Health and environmental protection applications	[239]
	CuNP_s_	Textile industry applications	[240]
		Biomedical applications	[241]
		Biofouling applications	[242]
		Water treatment applications	[243]
	ZnNP_s_	Heavy metal removal	[244]
		The performant adsorbent in water applications	[245]
	TiO_2_ NP_s_	Water treatment applications	[246]
		Anti-fouling applications	[247]
	Glycerol	Burn dressing applications	[248]
	PEG	Wound dressing applications	[249]
1D	CNTs	Wastewater treatment applications	[59]
	TiO_2_ CNT_s_	Proton-exchange membranes	[250]
	Attapulgite	Proton exchange membrane for fuel cells	[251]
	Cellulose/halloysite	Biomedical applications	[252]
2D	GO	The adsorbent in water purification	[212]
		Food industry	[253]
	SiO_2_@PVDF	Supercapacitor	[254]
		Membrane separation applications	[255]
	MMT	Drug delivery systems	[256]
		Food packing applications	[257]
	LAP@Ag NP_s_	Food packing applications	[258]

## Data Availability

Not available

## References

[1] Zubair M., Arshad M., Ullah A. (2020). Chitosan-Based Materials for Water and Wastewater Treatment. Handbook of Chitin and Chitosan.

[2] Shukla S.K., Mishra A.K., Arotiba O.A., Mamba B.B. (2013). Chitosan-based nanomaterials: A state-of-the-art review. Int. J. Biol. Macromol..

[3] Dodane V., Vilivalam V.D. (1998). Pharmaceutical applications of chitosan. Pharm. Sci. Technol. Today.

[4] Crini G., Badot P.-M. (2008). Application of chitosan, a natural aminopolysaccharide, for dye removal from aqueous solutions by adsorption processes using batch studies: A review of recent literature. Prog. Polym. Sci..

[5] Pan Y., Li Y., Zhao H., Zheng J., Xu H., Wei G., Hao J., Cui F. (2002). Bioadhesive polysaccharide in protein delivery system chitosan nanoparticles improve the intestinal absorption of insulin in vivo. Int. J. Pharm..

[6] Hirano S., Seino H., Akiyama Y., Nonaka I., Gebelein C.G., Dunn R.L. (1990). Chitosan: A Biocompatible Material for Oral and Intravenous Administrations. Progress in Biomedical Polymers.

[7] Muzzarelli R., Muzzarelli C. (2005). Chitosan Chemistry: Relevance to the Biomedical Sciences. Adv. Polym. Sci..

[8] Zhang H.-L., Wu S.-H., Tao Y., Zang L.-Q., Su Z.-Q. (2010). Preparation and Characterization of Water-Soluble Chitosan Nanoparticles as Protein Delivery System. J. Nanomater..

[9] Muxika A., Etxabide A., Uranga J., Guerrero P., de la Caba K. (2017). Chitosan as a bioactive polymer: Processing, properties and applications. Int. J. Biol. Macromol..

[10] Azuma K., Izumi R., Osaki T., Ifuku S., Morimoto M., Saimoto H., Minami S., Okamoto Y. (2015). Chitin, chitosan, and its derivatives for wound healing: Old and new materials. J. Funct. Biomater..

[11] Bedian L., Villalba-Rodriguez A.M., Hernandez-Vargas G., Parra-Saldivar R., Iqbal H.M. (2017). Bio-based materials with novel characteristics for tissue engineering applications—A review. Int. J. Biol. Macromol..

[12] Lizardi-Mendoza J., Monal W.M.A., Valencia F.M.G. (2016). Chemical Characteristics and Functional Properties of Chitosan. Chitosan in the Preservation of Agricultural Commodities.

[13] Younes I., Rinaudo M. (2015). Chitin and chitosan preparation from marine sources. Structure, properties and applications. Mar. Drugs.

[14] Mima S., Miya M., Iwamoto R., Yoshikawa S. (1983). Highly deacetylated chitosan and its properties. J. Appl. Polym. Sci..

[15] (2004). PubChem, 2D Structure Image of CID 71853. https://pubchem.ncbi.nlm.nih.gov/compound/71853.

[16] Suh J.K.F., Matthew H.W.T. (2000). Application of chitosan-based polysaccharide biomaterials in cartilage tissue engineering: A review. Biomaterials.

[17] Kumar M.N.V.R. (2000). A review of chitin and chitosan applications. React. Funct. Polym..

[18] Wang J., Zhuang S. (2018). Removal of various pollutants from water and wastewater by modified chitosan adsorbents. Crit. Rev. Environ. Sci. Technol..

[19] Zhang L., Zeng Y., Cheng Z. (2016). Removal of heavy metal ions using chitosan and modified chitosan: A review. J. Mol. Liq..

[20] Guibal E. (2004). Interactions of metal ions with chitosan-based sorbents: A review. Sep. Purif. Technol..

[21] Kuang S.P., Wang Z.Z., Liu J., Wu Z.C. (2013). Preparation of triethylene-tetramine grafted magnetic chitosan for adsorption of Pb(II) ion from aqueous solutions. J. Hazard. Mater..

[22] Kwok K.C., Koong L.F., Chen G., McKay G. (2014). Mechanism of arsenic removal using chitosan and nanochitosan. J. Colloid Interface Sci..

[23] Kumar A.S.K., Jiang S.-J. (2016). Chitosan-functionalized graphene oxide: A novel adsorbent an efficient adsorption of arsenic from aqueous solution. J. Environ. Chem. Eng..

[24] Northcott K.A., Snape I., Scales P.J., Stevens G.W. (2005). Dewatering behaviour of water treatment sludges associated with contaminated site remediation in Antarctica. Chem. Eng. Sci..

[25] Crini G. (2005). Recent developments in polysaccharide-based materials used as adsorbents in wastewater treatment. Prog. Polym. Sci..

[26] Suntornsuk W., Pochanavanich P., Suntornsuk L. (2002). Fungal chitosan production on food processing by-products. Process Biochem..

[27] Ham-Pichavant F., Sebe G., Pardon P., Coma V. (2005). Fat resistance properties of chitosan-based paper packaging for food applications. Carbohydr. Polym..

[28] Rinaudo M. (2006). Chitin and chitosan: Properties and applications. Prog. Polym. Sci..

[29] Chung Y.-C., Wang H.-L., Chen Y.-M., Li S.-L. (2003). Effect of abiotic factors on the antibacterial activity of chitosan against waterborne pathogens. Bioresour. Technol..

[30] Berger J., Reist M., Mayer J.M., Felt O., Gurny R. (2004). Structure and interactions in chitosan hydrogels formed by complexation or aggregation for biomedical applications. Eur. J. Pharm. Biopharm..

[31] Bodnar M., Hartmann J., Borbely J. (2005). Preparation and Characterization of Chitosan-Based Nanoparticles. Biomacromolecules.

[32] Ahmed S., Ikram S. (2016). Chitosan Based Scaffolds and Their Applications in Wound Healing. Achiev. Life Sci..

[33] Ardila N., Daigle F., Heuzey M.C., Ajji A. (2017). Antibacterial Activity of Neat Chitosan Powder and Flakes. Molecules.

[34] Wang Z., Lin S. (2017). Membrane fouling and wetting in membrane distillation and their mitigation by novel membranes with special wettability. Water Res..

[35] Salehi H., Rastgar M., Shakeri A. (2017). Anti-fouling and high water permeable forward osmosis membrane fabricated via layer by layer assembly of chitosan/graphene oxide. Appl. Surf. Sci..

[36] Jiang Y., Gong J.L., Zeng G.M., Ou X.M., Chang Y.N., Deng C.H., Zhang J., Liu H.Y., Huang S.Y. (2016). Magnetic chitosan-graphene oxide composite for anti-microbial and dye removal applications. Int. J. Biol. Macromol..

[37] Motshekga S.C., Ray S.S., Onyango M.S., Momba M.N.B. (2015). Preparation and antibacterial activity of chitosan-based nanocomposites containing bentonite-supported silver and zinc oxide nanoparticles for water disinfection. Appl. Clay Sci..

[38] Kamal T., Ul-Islam M., Khan S.B., Asiri A.M. (2015). Adsorption and photocatalyst assisted dye removal and bactericidal performance of ZnO/chitosan coating layer. Int. J. Biol. Macromol..

[39] Escudero-Oñate C., Martínez-Francés E. (2018). A Review of Chitosan-Based Materials for the Removal of Organic Pollution from Water and Bioaugmentation. Chitin-Chitosan—Myriad Functionalities in Science and Technology.

[40] Mourya V.K., Inamdar N.N. (2008). Chitosan-modifications and applications: Opportunities galore. React. Funct. Polym..

[41] Mirmohseni A., Dorraji M.S.S., Figoli A., Tasselli F. (2012). Chitosan hollow fibers as effective biosorbent toward dye: Preparation and modeling. Bioresour. Technol..

[42] Yang J., Lu H., Li M., Liu J., Zhang S., Xiong L., Sun Q. (2017). Development of chitosan-sodium phytate nanoparticles as a potent antibacterial agent. Carbohydr. Polym..

[43] Homez-Jara A., Daza L.D., Aguirre D.M., Munoz J.A., Solanilla J.F., Vaquiro H.A. (2018). Characterization of chitosan edible films obtained with various polymer concentrations and drying temperatures. Int. J. Biol. Macromol..

[44] Baker A.M., Cross W., Curtius K., Choi C.H.R., Al-Bakir I., Temko D., Martinez P., Williams M., Davis H., Biswas S. (2019). The evolutionary history of human colitis-associated colorectal cancer. Cancer Res..

[45] Kim T.-Y., Park S.-S., Cho S.-Y. (2012). Adsorption characteristics of Reactive Black 5 onto chitosan beads cross-linked with epichlorohydrin. J. Ind. Eng. Chem..

[46] Li X.-Q., Tang R.-C. (2016). Crosslinking of chitosan fiber by a water-soluble diepoxy crosslinker for enhanced acid resistance and its impact on fiber structures and properties. React. Funct. Polym..

[47] Poon L., Wilson L.D., Headley J.V. (2014). Chitosan-glutaraldehyde copolymers and their sorption properties. Carbohydr. Polym..

[48] Riegger B.R., Baurer B., Mirzayeva A., Tovar G.E.M., Bach M. (2018). A systematic approach of chitosan nanoparticle preparation via emulsion crosslinking as potential adsorbent in wastewater treatment. Carbohydr. Polym..

[49] Ngah W.S.W., Endud C.S., Mayanar R. (2002). Removal of copper (II) ions from aqueous solution onto chitosan and cross-linked chitosan beads. React. Funct. Polym..

[50] Prince J.A., Bhuvana S., Boodhoo K.V.K., Anbharasi V., Singh G. (2014). Synthesis and characterization of PEG-Ag immobilized PES hollow fiber ultrafiltration membranes with long lasting antifouling properties. J. Membr. Sci..

[51] Shi F., Ma Y., Ma J., Wang P., Sun W. (2012). Preparation and characterization of PVDF/TiO_2_ hybrid membranes with different dosage of nano-TiO_2_. J. Membr. Sci..

[52] Balta S., Sotto A., Luis P., Benea L., van der Bruggen B., Kim J. (2012). A new outlook on membrane enhancement with nanoparticles: The alternative of ZnO. J. Membr. Sci..

[53] García A., Rodríguez B., Oztürk D., Rosales M., Diaz D.I., Mautner A. (2017). Incorporation of CuO nanoparticles into thin-film composite reverse osmosis membranes (TFC-RO) for antibiofouling properties. Polym. Bull..

[54] Arsuaga J.M., Sotto A., Rosario G.l., Martınez A., Molina S., Teli S.B., Abajo J. (2013). Influence of the type, size, and distribution of metal oxide particleson the properties of nanocomposite ultrafiltration membranes. J. Membr. Sci..

[55] Yu S., Zuo X., Bao R., Xu X., Wang J., Xu J. (2009). Effect of SiO_2_ nanoparticle addition on the characteristics of a new organic–inorganic hybrid membrane. Polymer.

[56] Alam J., Alhoshan M., Dass L.A., Shukla A.K., Muthumareeswaran M.R., Hussain M., Aldwayyan A.S. (2016). Atomic layer deposition of TiO_2_ film on a polyethersulfone membrane: Separation applications. J. Polym. Res..

[57] Gzara L., Rehan Z.A., Khan S.B., Alamry K.A., Albeirutty M.H., El-Shahawi M.S., Rashid M.I., Figoli A., Drioli E., Asiri A.M. (2016). Preparation and characterization of PES-cobalt nanocomposite membranes with enhanced anti-fouling properties and performances. J. Taiwan Inst. Chem. E.

[58] Maximous N., Nakhla G., Wan W., Wong K. (2010). Performance of a novel ZrO_2_/PES membrane for wastewater filtration. J. Membr. Sci..

[59] Celik E., Park H., Choi H., Choi H. (2011). Carbon nanotube blended polyethersulfone membranes for fouling control in water treatment. Water Res..

[60] Xia S., Ni M. (2015). Preparation of poly(vinylidene fluoride) membranes with graphene oxide addition for natural organic matter removal. J. Membr. Sci..

[61] Fathizadeh M., Aroujalian A., Raisi A. (2011). Effect of added NaX nano-zeolite into polyamide as a top thin layer of membrane on water flux and salt rejection in a reverse osmosis process. J. Membr. Sci..

[62] Le N.L., Nunes S.P. (2016). Materials and membrane technologies for water and energy sustainability. Sustain. Mater. Technol..

[63] Liang S., Xiao K., Mo Y., Huang X. (2012). A novel ZnO nanoparticle blended polyvinylidene fluoride membrane for anti-irreversible fouling. J. Membr. Sci..

[64] Zhang X., Wanga Y., Liu Y., Xu J., Han Y., Xu X. (2014). Preparation, performances of PVDF-ZnO hybrid membranes and their applications in the removal of copper ions. Appl. Surf. Sci..

[65] Hong J., He Y. (2012). Effects of nano sized zinc oxide on the performance of PVDF microfiltration membranes. Desalination.

[66] Chung Y.T., Ba-Abbad M.M., Mohammad A.W., Benamor A. (2015). Functionalization of zinc oxide (ZnO) nanoparticles and its effects on polysulfone-ZnO membranes. Desalination Water Treat..

[67] Ghoul J.E., Ghiloufi I., L-Hobaib A.S.A., Mir L.E. (2017). Efficiency of polyamide thin-film nanocomposite membrane containing ZnO nanoparticles. J. Ovonic Res..

[68] Jo Y.J., Choi E.Y., Choi N.W., Kim C.K. (2016). Antibacterial and Hydrophilic Characteristics of Poly(ether sulfone) Composite Membranes Containing Zinc Oxide Nanoparticles Grafted with Hydrophilic Polymers. Ind. Eng. Chem. Res..

[69] Ursino C., Castro-Munoz R., Drioli E., Gzara L., Albeirutty M.H., Figoli A. (2018). Progress of Nanocomposite Membranes for Water Treatment. Membranes.

[70] Zhao S., Yan W., Shi M., Wang Z., Wang J., Wang S. (2015). Improving permeability and antifouling performance of polyethersulfone ultrafiltration membrane by incorporation of ZnO-DMF dispersion containing nano-ZnO and polyvinylpyrrolidone. J. Membr. Sci..

[71] Pintilie S., Tiron L.G., Bîrsan I., Balta S. (2017). Influence of ZnO Nanoparticle Size and Concentration on the Polysulfone Membrane Performance. Mater. Plast..

[72] Ronen A., Semiat R., Dosoretz C. (2013). Impact of ZnO embedded feed spacer on biofilm development in membrane systems. Water Res..

[73] Bai H., Liu Z., Sun D.D. (2012). A hierarchically structured and multifunctional membrane for water treatment. Appl. Catal. B Environ..

[74] Khan S.B., Alamry K.A., Bifari E.N., Asiri A.M., Yasir M., Gzara L., Ahmad R.Z. (2015). Assessment of antibacterial cellulose nanocomposites for water permeability and salt rejection. J. Ind. Eng. Chem..

[75] Akin I., Ersoz M. (2014). Preparation and characterization of CTA/m-ZnO composite membrane for transport of Rhodamine B. Desalination Water Treat..

[76] Ekambaram K., Doraisamy M. (2017). Surface modification of PVDF nanofiltration membrane using Carboxymethylchitosan-Zinc oxide bionanocomposite for the removal of inorganic salts and humic acid. Colloids Surf. A Physicochem. Eng. Asp..

[77] Li H., Shi W., Zhu H., Zhang Y., Du Q., Qin X. (2016). Effects of zinc oxide Nanospheres on the Separation performance of Hollow fiber poly(piperazine-amide) composite nanofiltration membranes. Fibers Polym..

[78] Zhao X., Li J., Liu C. (2017). Improving the separation performance of the forward osmosis membrane based on the etched microstructure of the supporting layer. Desalination.

[79] Isawi H., El-Sayed M.H., Feng X., Shawky H., Mottaleb M.S.A. (2016). Surface nanostructuring of thin film composite membranes via grafting polymerization and incorporation of ZnO nanoparticles. Appl. Surf. Sci..

[80] Zhang M., Zhang K., de Gusseme B., Verstraete W. (2012). Biogenic silver nanoparticles (bio-Ag 0) decrease biofouling of bio-Ag 0/PES nanocomposite membranes. Water Res..

[81] Alpatova A., Kim E.-S., Sun X., Hwang G., Liu Y., El-Din M.G. (2013). Fabrication of porous polymeric nanocomposite membranes with enhanced anti-fouling properties: Effect of casting composition. J. Membr. Sci..

[82] Rehan Z.A., Gzara L., Khan S.B., Alamry K.A., El-Shahawi M.S., Albeirutty M.H., Figoli A., Drioli E., Asiri A.M. (2016). Synthesis and Characterization of Silver Nanoparticles-Filled Polyethersulfone Membranes for Antibacterial and Anti-Biofouling Application. Recent Pat. Nanotechnol..

[83] Sile-Yuksel M., Tas B., Koseoglu-Imer D.Y., Koyuncu I. (2014). Effect of silver nanoparticle (AgNP) location in nanocomposite membrane matrix fabricated with different polymer type on antibacterial mechanism. Desalination.

[84] Koseoglu-Imer D.Y., Kose B., Altinbas M., Koyuncu I. (2013). The production of polysulfone (PS) membrane with silver nanoparticles (AgNP): Physical properties, filtration performances, and biofouling resistances of membranes. J. Membr. Sci..

[85] Hoek E.M.V., Ghosh A.K., Huang X., Liong M., Zink J.I. (2011). Physical–chemical properties, separation performance, and fouling resistance of mixed-matrix ultrafiltration membranes. Desalination.

[86] Escobar I.C., Bruggen B.V.d. (2015). Microfiltration and Ultrafiltration Membrane Science and Technology. J. Appl. Polym. Sci..

[87] Andrade P.F., de Faria A.F., Quites F.J., Oliveira S.R., Alves O.L., Arruda M.A.Z., Gonçalves M.d.C. (2015). Inhibition of bacterial adhesion on cellulose acetate membranes containing silver nanoparticles. Cellulose.

[88] Zhang Y., Wan Y., Shi Y., Pan G., Yan H., Xu J., Guo M., Qin L., Liu Y. (2016). Facile modification of thin-film composite nanofiltration membrane with silver nanoparticles for anti-biofouling. J. Polym. Res..

[89] Ben-Sasson M., Lu X., Bar-Zeev E., Zodrow K.R., Nejati S., Qi G., Giannelis E.P., Elimelech M. (2014). In situ formation of silver nanoparticles on thin-film composite reverse osmosis membranes for biofouling mitigation. Water Res..

[90] Yang Z., Wu Y., Wang J., Cao B., Tang C.Y. (2016). In Situ Reduction of Silver by Polydopamine: A Novel Antimicrobial Modification of a Thin-Film Composite Polyamide Membrane. Environ. Sci. Technol..

[91] Ahmad A., Jamshed F., Riaz T., Gul S.E., Waheed S., Sabir A., AlAnezi A.A., Adrees M., Jamil T. (2016). Self-sterilized composite membranes of cellulose acetate/polyethylene glycol for water desalination. Carbohydr. Polym..

[92] Liao Y., Wang R., Fane A.G. (2013). Engineering superhydrophobic surface on poly(vinylidene fluoride) nanofiber membranes for direct contact membrane distillation. J. Membr. Sci..

[93] Zhang S., Qiu G., Ting Y.P., Chung T.-S. (2013). Silver–PEGylated dendrimer nanocomposite coating for anti-fouling thin film composite membranes for water treatment. Colloids Surf. A Physicochem. Eng. Asp..

[94] Liu X., Foo L.-X., Li Y., Lee J.-Y., Cao B., Tang C.Y. (2016). Fabrication and characterization of nanocomposite pressure retarded osmosis (PRO) membranes with excellent anti-biofouling property and enhanced water permeability. Desalination.

[95] Zhang M., Field R.W., Zhang K. (2014). Biogenic silver nanocomposite polyethersulfone UF membranes with antifouling properties. J. Membr. Sci..

[96] Liu S., Fang F., Wu J., Zhang K. (2015). The anti-biofouling properties of thin-film composite nanofiltration membranes grafted with biogenic silver nanoparticles. Desalination.

[97] Liu S., Zhang M., Fang F., Cui L., Wu J., Field R., Zhang K. (2015). Biogenic silver nanocomposite TFC nanofiltration membrane with antifouling properties. Desalination Water Treat..

[98] Akar N., Asar B., Dizge N., Koyuncu I. (2013). Investigation of characterization and biofouling properties of PES membrane containing selenium and copper nanoparticles. J. Membr. Sci..

[99] Ben-Sasson M., Zodrow K.R., Genggeng Q., Kang Y., Giannelis E.P., Elimelech M. (2014). Surface functionalization of thin-film composite membranes with copper nanoparticles for antimicrobial surface properties. Environ. Sci. Technol..

[100] Madaeni S.S., Zinadini S., Vatanpour V. (2011). A new approach to improve antifouling property of PVDF membrane using in situ polymerization of PAA functionalized TiO_2_ nanoparticles. J. Membr. Sci..

[101] Teow Y.H., Ooi B.S., Ahmad A.L., Lim J.K. (2012). Mixed-Matrix Membrane for Humic Acid Removal: Influence of Different Types of TiO_2_ on Membrane Morphology and Performance. Int. J. Chem. Eng. Appl..

[102] Rajaeian B., Heitz A., Tade M.O., Liu S. (2015). Improved separation and antifouling performance of PVA thin film nanocomposite membranes incorporated with carboxylated TiO_2_ nanoparticles. J. Membr. Sci..

[103] Méricq J.P., Mendret J., Brosillon S., Faur C. (2015). High performance PVDF-TiO_2_ membranes for water treatment. Chem. Eng. Sci..

[104] Mollahosseini A., Rahimpour A. (2014). Interfacially polymerized thin film nanofiltration membranes on TiO_2_ coated polysulfone substrate. J. Ind. Eng. Chem..

[105] Abedini R., Mousavi S.M., Aminzadeh R. (2011). A novel cellulose acetate (CA) membrane using TiO_2_ nanoparticles: Preparation, characterization and permeation study. Desalination.

[106] Ngo T.H.A., Nguyen D.T., Do K.D., Nguyen T.T.M., Mori S., Tran D.T. (2016). Surface modification of polyamide thin film composite membrane by coating of titanium dioxide nanoparticles. J. Sci. Adv. Mater. Devices.

[107] Kim S.-J., Lee P.-S., Bano S., Park Y.-I., Nam S.-E., Lee K.-H. (2016). Effective incorporation of TiO_2_ nanoparticles into polyamide thin-film composite membranes. J. Appl. Polym. Sci..

[108] Amini M., Rahimpour A., Jahanshahi M. (2015). Forward osmosis application of modified TiO_2_-polyamide thin film nanocomposite membranes. Desalination Water Treat..

[109] Moghadam F., Omidkhah M.R., Vasheghani-Farahani E., Pedram M.Z., Dorosti F. (2011). The effect of TiO_2_ nanoparticles on gas transport properties of Matrimid5218-based mixed matrix membranes. Sep. Purif. Technol..

[110] Hu W., Yin J., Deng B., Hu Z. (2015). Application of nano TiO_2_ modified hollow fiber membranes in algal membrane bioreactors for high-density algae cultivation and wastewater polishing. Bioresour. Technol..

[111] Sotto A., Boromand A., Balta S., Kim J., van der Bruggen B. (2011). Doping of polyethersulfone nanofiltration membranes: Antifouling effect observed at ultralow concentrations of TiO_2_ nanoparticles. J. Mater. Chem..

[112] Kim E.-S., Hwang G., El-Din M.G., Liu Y. (2012). Development of nanosilver and multi-walled carbon nanotubes thin-film nanocomposite membrane for enhanced water treatment. J. Membrane Sci..

[113] Ahmad F., Santos C.M., Mangadlao J., Advincula R., Rodrigues D.F. (2013). Antimicrobial PVK:SWNT nanocomposite coated membrane for water purification: Performance and toxicity testing. Water Res..

[114] Daraei P., Madaeni S.S., Ghaemi N., Khadivi M.A., Astinchap B., Moradian R. (2013). Enhancing antifouling capability of PES membrane via mixing with various types of polymer modified multi-walled carbon nanotube. J. Membr. Sci..

[115] Kim E.-S., Liu Y., El-Din M.G. (2013). An in-situ integrated system of carbon nanotubes nanocomposite membrane for oil sands process-affected water treatment. J. Membr. Sci..

[116] Shah P., Murthy C.N. (2013). Studies on the porosity control of MWCNT/polysulfone composite membrane and its effect on metal removal. J. Membr. Sci..

[117] Shen J.n., Yu C.c., Ruan H.m., Gao C.j., van der Bruggen B. (2013). Preparation and characterization of thin-film nanocomposite membranes embedded with poly(methyl methacrylate) hydrophobic modified multiwalled carbon nanotubes by interfacial polymerization. J. Membr. Sci..

[118] Grosso V., Vuono D., Bahattab M.A., di Profio G., Curcio E., Al-Jilil S.A., Alsubaie F., Alfife M., Nagy J.B., Drioli E. (2014). Polymeric and mixed matrix polyimide membranes. Sep. Purif. Technol..

[119] Sianipar M., Kim S.H., Min C., Tijing L.D., Shon H.K. (2016). Potential and performance of a polydopamine-coated multiwalled carbon nanotube/polysulfone nanocomposite membrane for ultrafiltration application. J. Ind. Eng. Chem..

[120] Khalid A., Abdel-Karim A., Atieh M.A., Javed S., McKay G. (2018). PEG-CNTs nanocomposite PSU membranes for wastewater treatment by membrane bioreactor. Sep. Purif. Technol..

[121] Mulopo J. (2017). Bleach plant effluent treatment in anaerobic membrane bioreactor (AMBR) using carbon nanotube/polysulfone nanocomposite membranes. J. Environ. Chem. Eng..

[122] Zhao C., Xu X., Chen J., Yang F. (2014). Optimization of preparation conditions of poly(vinylidene fluoride)/graphene oxide microfiltration membranes by the Taguchi experimental design. Desalination.

[123] Xia S., Yao L., Zhao Y., Li N., Zheng Y. (2015). Preparation of graphene oxide modified polyamide thin film composite membranes with improved hydrophilicity for natural organic matter removal. Chem. Eng. J..

[124] Lee J., Chae H.-R., Won Y.J., Lee K., Lee C.-H., Lee H.H., Kim I.-C., Lee J.-M. (2013). Graphene oxide nanoplatelets composite membrane with hydrophilic and antifouling properties for wastewater treatment. J. Membr. Sci..

[125] Pastrana-Martinez L.M., Morales-Torres S., Figueiredo J.L., Faria J.L., Silva A.M.T. (2015). Graphene oxide based ultrafiltration membranes for photocatalytic degradation of organic pollutants in salty water. Water Res..

[126] Kiran S.A., Thuyavan Y.L., Arthanareeswaran G., Matsuura T., Ismail A.F. (2016). Impact of graphene oxide embedded polyethersulfone membranes for the effective treatment of distillery effluent. Chem. Eng. J..

[127] Ganesh B.M., Isloor A.M., Ismail A.F. (2013). Enhanced hydrophilicity and salt rejection study of graphene oxide-polysulfone mixed matrix membrane. Desalination.

[128] Goh K., Setiawan L., Wei L., Si R., Fane A.G., Wang R., Chen Y. (2015). Graphene oxide as effective selective barriers on a hollow fiber membrane for water treatment process. J. Membr. Sci..

[129] Zinadini S., Zinatizadeh A.A., Rahimi M., Vatanpour V., Zangeneh H. (2014). Preparation of a novel antifouling mixed matrix PES membrane by embedding graphene oxide nanoplates. J. Membr. Sci..

[130] Wang J., Zhao C., Wang T., Wu Z., Li X., Li J. (2016). Graphene oxide polypiperazine-amide nanofiltration membrane for improving flux and anti-fouling in water purification. RSC Adv..

[131] Chae H.-R., Lee J., Lee C.-H., Kim I.-C., Park P.-K. (2015). Graphene oxide-embedded thin-film composite reverse osmosis membrane with high flux, anti-biofouling, and chlorine resistance. J. Membr. Sci..

[132] He L., Dumée L.F., Feng C., Velleman L., Reis R., She F., Gao W., Kong L. (2015). Promoted water transport across graphene oxide–poly(amide) thin film composite membranes and their antibacterial activity. Desalination.

[133] Shen L., Xiong S., Wang Y. (2016). Graphene oxide incorporated thin-film composite membranes for forward osmosis applications. Chem. Eng. Sci..

[134] Crock C.A., Rogensues A.R., Shan W., Tarabara V.V. (2013). Polymer nanocomposites with graphene-based hierarchical fillers as materials for multifunctional water treatment membranes. Water Res..

[135] Han Y., Xu Z., Gao C. (2013). Ultrathin Graphene Nanofiltration Membrane for Water Purification. Adv. Funct. Mater..

[136] Alzahrani S., Mohammad A.W. (2014). Challenges and trends in membrane technology implementation for produced water treatment: A review. J. Water Process Eng..

[137] Bruggen B.V., Lejon L., Vandecasteele C. (2003). Reuse, treatment, and discharge of the concentrate of pressure-driven membrane processes. Environ. Sci. Technol..

[138] Kim J., van der Bruggen B. (2010). The use of nanoparticles in polymeric and ceramic membrane structures: Review of manufacturing procedures and performance improvement for water treatment. Environ. Pollut..

[139] Castro-Munoz R., Yanez-Fernandez J., Fila V. (2016). Phenolic compounds recovered from agro-food by-products using membrane technologies: An overview. Food Chem..

[140] Lalia B.S., Kochkodan V., Hashaikeh R., Hilal N. (2013). A review on membrane fabrication: Structure, properties and performance relationship. Desalination.

[141] Ulbricht M. (2006). Advanced functional polymer membranes. Polymer.

[142] Ng L.Y., Mohammad A.W., Leo C.P., Hilal N. (2013). Polymeric membranes incorporated with metal/metal oxide nanoparticles: A comprehensive review. Desalination.

[143] Bakar N.H.H.A., Tan W.L., Ahmad M.I., Ismail M., Riffat S. (2016). Natural Composite Membranes for Water Remediation: Toward a Sustainable Tomorrow. Renewable Energy and Sustainable Technologies for Building and Environmental Applications: Options for a Greener Future.

[144] Nackaerts R. (2014). Are Membranes Implemented with Nanoparticles Able to Provide a Breakthrough in Water Purification.

[145] Flemming H.-C. (1997). Reverse osmosis membrane biofouling. Exp. Therm. Fluid Sci..

[146] Boussu K., Belpaire A., Volodin A., van Haesendonck C., van der Meeren P., Vandecasteele C., van der Bruggen B. (2007). Influence of membrane and colloid characteristics on fouling of nanofiltration membranes. J. Membr. Sci..

[147] Mohammad A.W., Hilal N., Seman M.N.A. (2003). A study on producing composite nanofiltration membranes with optimized properties. Desalination.

[148] Robeson L.M. (1991). Correlation of separation factor versus permeability for polymeric membranes. J. Membr. Sci..

[149] al Aani S., Wright C.J., Atieh M.A., Hilal N. (2017). Engineering nanocomposite membranes: Addressing current challenges and future opportunities. Desalination.

[150] Pendergast M.M., Hoek E.M.V. (2011). A review of water treatment membrane nanotechnologies. Energy Environ. Sci..

[151] Mueller N.C., van der Bruggen B., Keuter V., Luis P., Melin T., Pronk W., Reisewitz R., Rickerby D., Rios G.M., Wennekes W. (2012). Nanofiltration and nanostructured membranes--should they be considered nanotechnology or not?. J. Hazard. Mater..

[152] Marino T., Boerrigter M., Faccini M., Chaumette C., Arockiasamy L., Bundschuh J., Figoli A. (2017). Photocatalytic Activity and Synthesis Procedures of TiO_2_ Nanoparticles for Potential Applications in Membranes. Application of Nanotechnology in Membranes for Water Treatment.

[153] Madaeni S.S., Ghaemi N., Rajabi H. (2015). Advances in Polymeric Membranes for Water Treatment.

[154] Vatanpour V., Madaeni S.S., Khataee A.R., Salehi E., Zinadini S., Monfared H.A. (2012). TiO_2_ embedded mixed matrix PES nanocomposite membranes: Influence of different sizes and types of nanoparticles on antifouling and performance. Desalination.

[155] Park J.Y., Lee C., Jung K.W., Jung D. (2009). Structure Related Photocatalytic Properties of TiO_2_. Bull. Korean Chem. Soc..

[156] Sotto A., Boromand A., Zhang R., Luis P., Arsuaga J.M., Kim J., van der Bruggen B. (2011). Effect of nanoparticle aggregation at low concentrations of TiO_2_ on the hydrophilicity, morphology, and fouling resistance of PES-TiO_2_ membranes. J. Colloid Interface Sci..

[157] Zhang R.-X., Braeken L., Luis P., Wang X.-L., van der Bruggen B. (2013). Novel binding procedure of TiO_2_ nanoparticles to thin film composite membranes via self-polymerized polydopamine. J. Membr. Sci..

[158] Zhang C., Huang M., Meng L., Li B., Cai T. (2017). Electrospun polysulfone (PSf)/titanium dioxide (TiO_2_) nanocomposite fibers as substrates to prepare thin film forward osmosis membranes. J. Chem. Technol. Biotechnol..

[159] Zapata P.A., Larrea M., Tamayo L., Rabagliati F.M., Azocar M.I., Paez M. (2016). Polyethylene/silver-nanofiber composites: A material for antibacterial films. Mater. Sci. Eng. C Mater. Biol. Appl..

[160] Graham N. (1998). Guidelines for Drinking-Water Quality.

[161] Chaloupka K., Malam Y., Seifalian A.M. (2010). Nanosilver as a new generation of nanoproduct in biomedical applications. Trends Biotechnol..

[162] Wei L., Lu J., Xu H., Patel A., Chen Z.-S., Chen G. (2015). Silver nanoparticles: Synthesis, properties, and therapeutic applications. Drug Discov. Today.

[163] Guo L., Yuan W., Lu Z., Li C.M. (2013). Polymer/nanosilver composite coatings for antibacterial applications. Colloids Surf. A Physicochem. Eng. Asp..

[164] Cao X., Tang M., Liu F., Nie Y., Zhao C. (2010). Immobilization of silver nanoparticles onto sulfonated polyethersulfone membranes as antibacterial materials. Colloids Surf. B Biointerfaces.

[165] Zhu X., Bai R., Wee K.-H., Liu C., Tang S.-L. (2010). Membrane surfaces immobilized with ionic or reduced silver and their anti-biofouling performances. J. Membr. Sci..

[166] Haider M.S., Shao G.N., Imran S.M., Park S.S., Abbas N., Tahir M.S., Hussain M., Bae W., Kim H.T. (2016). Aminated polyethersulfone-silver nanoparticles (AgNPs-APES) composite membranes with controlled silver ion release for antibacterial and water treatment applications. Mater. Sci. Eng. C.

[167] Biswas P., Bandyopadhyaya R. (2017). Biofouling prevention using silver nanoparticle impregnated polyethersulfone (PES) membrane: *E. coli* cell-killing in a continuous cross-flow membrane module. J. Colloid Interf. Sci..

[168] Andrade P.F., de Faria A.F., Oliveira S.R., Arruda M.A.Z., Gonçalves M.d.C. (2015). Improved antibacterial activity of nanofiltration polysulfone membranes modified with silver nanoparticles. Water Res..

[169] Ruparelia J.P., Chatterjee A.K., Duttagupta S.P., Mukherji S. (2008). Strain specificity in antimicrobial activity of silver and copper nanoparticles. Acta Biomater..

[170] Yoon K.-Y., Byeon J.H., Park J.-H., Hwang J. (2007). Susceptibility constants of Escherichia coli and Bacillus subtilis to silver and copper nanoparticles. Sci. Total Environ..

[171] Varkey A.J., Dlamini M.D. (2012). Point-of-use water purification using clay pot water filters and copper mesh. Water SA.

[172] Ren G., Hu D., Cheng E.W.C., Vargas-Reus M.A., Reip P., Allaker R.P. (2009). Characterisation of copper oxide nanoparticles for antimicrobial applications. Int. J. Antimicrob. Agents.

[173] Tamayo L., Azócar M., Kogan M., Riveros A., Páez M. (2016). Copper-polymer nanocomposites: An excellent and cost-effective biocide for use on antibacterial surfaces. Mater. Sci. Eng. C.

[174] Modrzejewska Z., Rogacki G., Sujka W., Zarzycki R. (2016). Sorption of copper by chitosan hydrogel: Kinetics and equilibrium. Chem. Eng. Process. Process Intensif..

[175] Boamah P.O., Huang Y., Hua M., Onumah J., Sam-Amoah L.K., Boamah P.O., Qian Y., Zhang Q. (2016). Sorption of copper onto low molecular weight chitosan derivative from aqueous solution. Ecotoxicol. Environ. Saf..

[176] Ghasemzadeh G., Momenpour M., Omidi F., Hosseini M.R., Ahani M., Barzegari A. (2014). Applications of nanomaterials in water treatment and environmental remediation. Front. Environ. Sci. Eng..

[177] Shao W., Wang S., Wu J., Huang M., Liu H., Min H. (2016). Synthesis and antimicrobial activity of copper nanoparticle loaded regenerated bacterial cellulose membranes. RSC Adv..

[178] Hausman R., Gullinkala T., Escobar I.C. (2010). Development of copper-charged polypropylene feedspacers for biofouling control. J. Membr. Sci..

[179] Araújo P.A., Miller D.J., Correia P.B., van Loosdrecht M.C.M., Kruithof J.C., Freeman B.D., Paul D.R., Vrouwenvelder J.S. (2012). Impact of feed spacer and membrane modification by hydrophilic, bactericidal and biocidal coating on biofouling control. Desalination.

[180] Motelica L., Ficai D., Ficai A., Oprea O.C., Kaya D.A., Andronescu E. (2020). Biodegradable Antimicrobial Food Packaging: Trends and Perspectives. Foods.

[181] Xu J., Feng X., Chen P., Gao C. (2012). Development of an antibacterial copper (II)-chelated polyacrylonitrile ultrafiltration membrane. J. Membr. Sci..

[182] Xu J., Zhang L., Gao X., Bie H., Fu Y., Gao C. (2015). Constructing antimicrobial membrane surfaces with polycation–copper(II) complex assembly for efficient seawater softening treatment. J. Membr. Sci..

[183] Baruah S., Pal S.K., Dutta J. (2012). Nanostructured Zinc Oxide for Water Treatment. Nanosci. Nanotechnol. Asia.

[184] Leo C.P., Lee W.P.C., Ahmad A.L., Mohammad A.W. (2012). Polysulfone membranes blended with ZnO nanoparticles for reducing fouling by oleic acid. Sep. Purif. Technol..

[185] Shen L., Bian X., Lu X., Shi L., Liu Z., Chen L., Hou Z., Fan K. (2012). Preparation and characterization of ZnO/polyethersulfone (PES) hybrid membranes. Desalination.

[186] Lin W., Xu Y., Huang C.-C., Ma Y., Shannon K.B., Chen D.-R., Huang Y.-W. (2009). Toxicity of nano- and micro-sized ZnO particles in human lung epithelial cells. J. Nanopart Res..

[187] Elmi F., Alinezhad H., Moulana Z., Salehian F., Tavakkoli S.M., Asgharpour F., Fallah H., Elmi M.M. (2014). The use of antibacterial activity of ZnO nanoparticles in the treatment of municipal wastewater. Water Sci. Technol..

[188] Jhaveri J.H., Murthy Z.V.P. (2016). Nanocomposite membranes. Desalination Water Treat..

[189] Wang Y., Yang L., Luo G., Dai Y. (2009). Preparation of cellulose acetate membrane filled with metal oxide particles for the pervaporation separation of methanol/methyl tert-butyl ether mixtures. Chem. Eng. J..

[190] Saleh T.A., Gupta V.K. (2012). Synthesis and characterization of alumina nano-particles polyamide membrane with enhanced flux rejection performance. Sep. Purif. Technol..

[191] Mojtahedi Y.M., Mehrnia M.R., Homayoonfal M. (2013). Fabrication of Al_2_O_3_/PSf nanocomposite membranes: Efficiency comparison of coating and blending methods in modification of filtration performance. Desalination Water Treat..

[192] Dong H., Xiao K.-j., Li X.-l., Ren Y., Guo S.-Y. (2013). Preparation of PVDF/Al_2_O_3_ hybrid membrane via the sol–gel process and characterization of the hybrid membrane. Desalination Water Treat..

[193] Homayoonfal M., Mehrnia M.R., Rahmani S., Mojtahedi Y.M. (2015). Fabrication of alumina/polysulfone nanocomposite membranes with biofouling mitigation approach in membrane bioreactors. J. Ind. Eng. Chem..

[194] Ma B., Hu C., Wang X., Xie Y., Jefferson W.A., Liu H., Qu J. (2015). Effect of aluminum speciation on ultrafiltration membrane fouling by low dose aluminum coagulation with bovine serum albumin (BSA). J. Membr. Sci..

[195] Niksefat N., Jahanshahi M., Rahimpour A. (2014). The effect of SiO_2_ nanoparticles on morphology and performance of thin film composite membranes for forward osmosis application. Desalination.

[196] Kebria M.R.S., Jahanshahi M., Rahimpour A. (2015). SiO_2_ modified polyethyleneimine-based nanofiltration membranes for dye removal from aqueous and organic solutions. Desalination.

[197] Homaeigohar S. (2020). Water Treatment with New Nanomaterials. Water.

[198] Ghadimi M., Zangenehtabar S., Homaeigohar S. (2020). An Overview of the Water Remediation Potential of Nanomaterials and Their Ecotoxicological Impacts. Water.

[199] Fontananova E., Grosso V., Aljlil S.A., Bahattab M.A., Vuono D., Nicoletta F.P., Curcio E., Drioli E., di Profio G. (2017). Effect of functional groups on the properties of multiwalled carbon nanotubes/polyvinylidenefluoride composite membranes. J. Membr. Sci..

[200] Homaeigohar S., Zillohu A.U., Abdelaziz R., Hedayati M.K., Elbahri M. (2016). A Novel Nanohybrid Nanofibrous Adsorbent for Water Purification from Dye Pollutants. Materials.

[201] Homaeigohar S.S., Buhr K., Ebert K. (2010). Polyethersulfone electrospun nanofibrous composite membrane for liquid filtration. J. Membr. Sci..

[202] Homaeigohar S.S., Elbahri M. (2012). Novel compaction resistant and ductile nanocomposite nanofibrous microfiltration membranes. J. Colloid Interf. Sci..

[203] Homaeigohar S.S., Mahdavi H., Elbahri M. (2012). Extraordinarily water permeable sol–gel formed nanocomposite nanofibrous membranes. J. Colloid Interf. Sci..

[204] Pan S.-F., Ke X.-X., Wang T.-Y., Liu Q., Zhong L.-B., Zheng Y.-M. (2019). Synthesis of Silver Nanoparticles Embedded Electrospun PAN Nanofiber Thin-Film Composite Forward Osmosis Membrane to Enhance Performance and Antimicrobial Activity. Ind. Eng. Chem. Res..

[205] Hofs B., Schurer R., Harmsen D.J.H., Ceccarelli C., Beerendonk E.F., Cornelissen E.R. (2013). Characterization and performance of a commercial thin film nanocomposite seawater reverse osmosis membrane and comparison with a thin film composite. J. Membr. Sci..

[206] Kim W.-g., Nair S. (2013). Membranes from nanoporous 1D and 2D materials: A review of opportunities, developments, and challenges. Chem. Eng. Sci..

[207] Gugliuzza A., Politano A., Drioli E. (2017). The advent of graphene and other two-dimensional materials in membrane science and technology. Curr. Opin. Chem. Eng..

[208] Aghigh A., Alizadeh V., Wong H.Y., Islam M.S., Amin N., Zaman M. (2015). Recent advances in utilization of graphene for filtration and desalination of water: A review. Desalination.

[209] Gao P., Tai M.H., Sun D.D. (2013). Hierarchical TiO_2_/V_2_O_5_ Multifunctional Membrane for Water Purification. Chempluschem.

[210] Ionita M., Pandele A.M., Crica L., Pilan L. (2014). Improving the thermal and mechanical properties of polysulfone by incorporation of graphene oxide. Compos. Part B Eng..

[211] Jhaveri J.H., Murthy Z.V.P. (2016). A comprehensive review on anti-fouling nanocomposite membranes for pressure driven membrane separation processes. Desalination.

[212] Croitoru A.M., Ficai A., Ficai D., Trusca R., Dolete G., Andronescu E., Turculet S.C. (2020). Chitosan/Graphene Oxide Nanocomposite Membranes as Adsorbents with Applications in Water Purification. Materials.

[213] Sophia A.C., Lima E.C., Allaudeen N., Rajan S. (2016). Application of graphene based materials for adsorption of pharmaceutical traces from water and wastewater—A review. Desalination Water Treat..

[214] Yang M., Zhao C., Zhang S., Li P., Hou D. (2017). Preparation of graphene oxide modified poly(m-phenylene isophthalamide) nanofiltration membrane with improved water flux and antifouling property. Appl. Surf. Sci..

[215] Xu C., Cui A., Xu Y., Fu X. (2013). Graphene oxide–TiO_2_ composite filtration membranes and their potential application for water purification. Carbon.

[216] Kochameshki M.G., Marjani A., Mahmoudian M., Farhadi K. (2017). Grafting of diallyldimethylammonium chloride on graphene oxide by RAFT polymerization for modification of nanocomposite polysulfone membranes using in water treatment. Chem. Eng. J..

[217] Liu Q., Xu G.-R. (2016). Graphene oxide (GO) as functional material in tailoring polyamide thin film composite (PA-TFC) reverse osmosis (RO) membranes. Desalination.

[218] Yin J., Zhu G., Deng B. (2016). Graphene oxide (GO) enhanced polyamide (PA) thin-film nanocomposite (TFN) membrane for water purification. Desalination.

[219] Ali M.E.A., Wang L., Wang X., Feng X. (2016). Thin film composite membranes embedded with graphene oxide for water desalination. Desalination.

[220] Yang G.C.C., Chen Y.-C., Yang H.-X., Yen C.-H. (2016). Performance and mechanisms for the removal of phthalates and pharmaceuticals from aqueous solution by graphene-containing ceramic composite tubular membrane coupled with the simultaneous electrocoagulation and electrofiltration process. Chemosphere.

[221] Xiaowei Q., Na L., Qinzhuo W., Ji S. (2018). Chitosan/graphene oxide mixed matrix membrane with enhanced water permeability for high-salinity water desalination by pervaporation. Desalination.

[222] Deng H., Sun P., Zhang Y., Zhu H. (2016). Reverse osmosis desalination of chitosan cross-linked graphene oxide/titania hybrid lamellar membranes. Nanotechnology.

[223] Garofalo A., Donato L., Drioli E., Criscuoli A., Carnevale M.C., Alharbi O., Aljlil S.A., Algieri C. (2014). Supported MFI zeolite membranes by cross flow filtration for water treatment. Sep. Purif. Technol..

[224] Garofalo A., Carnevale M.C., Donato L., Drioli E., Alharbi O., Aljlil S.A., Criscuoli A., Algieri C. (2016). Scale-up of MFI zeolite membranes for desalination by vacuum membrane distillation. Desalination.

[225] Zhu B., Myat D.T., Shin J.-W., Na Y.-H., Moon I.-S., Connor G., Maeda S., Morris G., Gray S., Duke M. (2015). Application of robust MFI-type zeolite membrane for desalination of saline wastewater. J. Membr. Sci..

[226] Swenson P., Tanchuk B., Gupta A., An W., Kuznicki S.M. (2012). Pervaporative desalination of water using natural zeolite membranes. Desalination.

[227] Gascon J., Kapteijn F., Zornoza B., Sebastián V., Casado C., Coronas J. (2012). Practical Approach to Zeolitic Membranes and Coatings: State of the Art, Opportunities, Barriers, and Future Perspectives. Chem. Mater..

[228] Huang H., Qu X., Ji X., Gao X., Zhang L., Chen H., Hou L. (2013). Acid and multivalent ion resistance of thin film nanocomposite RO membranes loaded with silicalite-1 nanozeolites. J. Mater. Chem. A.

[229] Dong L.-x., Huang X.-c., Wang Z., Yang Z., Wang X.-m., Tang C.Y. (2016). A thin-film nanocomposite nanofiltration membrane prepared on a support with in situ embedded zeolite nanoparticles. Sep. Purif. Technol..

[230] Kumar S., Prasad K., Gil J.M., Sobral A., Koh J. (2018). Mesoporous zeolite-chitosan composite for enhanced capture and catalytic activity in chemical fixation of CO_2_. Carbohydr. Polym..

[231] Ahmad A.L., Majid M.A., Ooi B.S. (2011). Functionalized PSf/SiO_2_ nanocomposite membrane for oil-in-water emulsion separation. Desalination.

[232] Thakur M.K., Gupta R.K., Thakur V.K. (2014). Surface modification of cellulose using silane coupling agent. Carbohydr. Polym..

[233] Lim S.-R., Schoenung J.M. (2010). Human health and ecological toxicity potentials due to heavy metal content in waste electronic devices with flat panel displays. J. Hazard. Mater..

[234] Karlsson K., Viklander M., Scholes L., Revitt M. (2010). Heavy metal concentrations and toxicity in water and sediment from stormwater ponds and sedimentation tanks. J. Hazard. Mater..

[235] Madoni P., Romeo M.G. (2006). Acute toxicity of heavy metals towards freshwater ciliated protists. Environ. Pollut..

[236] Aizat M.A., Aziz F., Ahsan A., Ismail A.F. (2019). 12—Chitosan Nanocomposite Application in Wastewater Treatments. Nanotechnology in Water and Wastewater Treatment.

[237] Sharma R., Kumar D. (2019). Chitosan-Based Membranes for Wastewater Desalination and Heavy Metal Detoxification. Nanoscale Mater. Water Purif..

[238] Li J., Gao J., Sui G., Jia L., Zuo C., Deng Q. (2014). Influence of a glycerin additive on the structure and water vapor permeance of chitosan membranes. Mater. Express.

[239] Khaydarov R., Khaydarov R., Evgrafova S., Wagner S., Cho S. (1970). Environmental and Human Health Issues of Silver Nanoparticles Applications. Environmental Security and Ecoterrorism.

[240] Xu Q., Ke X., Ge N., Shen L., Zhang Y., Fu F., Liu X. (2018). Preparation of Copper Nanoparticles Coated Cotton Fabrics with Durable Antibacterial Properties. Fibers Polym..

[241] Verma N., Kumar N. (2019). Synthesis and Biomedical Applications of Copper Oxide Nanoparticles: An Expanding Horizon. ACS Biomater. Sci. Eng..

[242] Chapman J., Nor L., Brown R., Kitteringham E., Russell S., Sullivan T., Regan F. (2013). Antifouling performances of macro- to micro- to nano-copper materials for the inhibition of biofouling in its early stages. J. Mater. Chem. B.

[243] Dankovich T., Smith J. (2014). Incorporation of copper nanoparticles into paper for point-of-use water purification. Water Res..

[244] Le A.T., Pung S.-Y., Sreekantan S., Matsuda A., Huynh D.P. (2019). Mechanisms of removal of heavy metal ions by ZnO particles. Heliyon.

[245] Nalwa K., Thakur A., Sharma N. (2017). Synthesis of ZnO nanoparticles and its application in adsorption. Adv. Mater..

[246] Acheampong M., Antwi D. (2016). Modification of Titanium Dioxide for Wastewater Treatment Application and Its Recovery for Reuse. J. Environ. Sci. Eng. A.

[247] Zhang R.-X., Braeken L., Liu T.-Y., Luis P., Wang X.-L., van der Bruggen B. (2017). Remarkable Anti-Fouling Performance of TiO_2_-Modified TFC Membranes with Mussel-Inspired Polydopamine Binding. Appl. Sci..

[248] Khoo T.L., Halim A.S., Saad A.M., Dorai A.A. (2010). The application of glycerol-preserved skin allograft in the treatment of burn injuries: An analysis based on indications. Burns.

[249] Chen S.L., Fu R.H., Liao S.F., Liu S.P., Lin S.Z., Wang Y.C. (2018). A PEG-Based Hydrogel for Effective Wound Care Management. Cell Transpl..

[250] Tsen W.C. (2020). Hydrophilic TiO_2_ decorated carbon nanotubes/sulfonated poly(ether ether ketone) composite proton exchange membranes for fuel cells. Polym. Eng. Sci..

[251] Hu F., Li T., Zhong F., Wen S., Zheng G., Gong C., Qin C., Liu H. (2020). Preparation and properties of chitosan/acidified attapulgite composite proton exchange membranes for fuel cell applications. J. Appl. Polym. Sci..

[252] Sharifzadeh G., Soheilmoghaddam M., Adelnia H., Wahit M.U., Arzhandi M.R.D., Moslehyani A. (2020). Biocompatible regenerated cellulose/halloysite nanocomposite fibers. Polym. Eng. Sci..

[253] Sundramoorthy A., Gunasekaran S. (2014). Applications of graphene in quality assurance and safety of food. TRAC Trends Anal. Chem..

[254] Ortega P., Trigueiro J., Silva G., Lavall R. (2015). Improving supercapacitor capacitance by using a novel gel nanocomposite polymer electrolyte based on nanostructured SiO_2_, PVDF and imidazolium ionic liquid. Electrochim. Acta.

[255] Yu L.-Y., Xu Z.-L., Shen H.-M., Yang H. (2009). Preparation and characterization of PVDF–SiO_2_ composite hollow fiber UF membrane by sol–gel method. J. Membr. Sci..

[256] Abdeen R., Salahuddin N. (2013). Modified Chitosan-Clay Nanocomposite as a Drug Delivery System Intercalation andIn VitroRelease of Ibuprofen. J. Chem-Ny.

[257] Kasirga Y., Oral A., Caner C. (2012). Preparation and characterization of chitosan/montmorillonite-K10 nanocomposites films for food packaging applications. Polym. Compos..

[258] Wu Z.G., Huang X.J., Li Y.C., Xiao H.Z., Wang X.Y. (2018). Novel chitosan films with laponite immobilized Ag nanoparticles for active food packaging. Carbohydr. Polym..

[259] Murthy Z.V.P., Chaudhari L.B. (2008). Application of nanofiltration for the rejection of nickel ions from aqueous solutions and estimation of membrane transport parameters. J. Hazard. Mater..

[260] Nanda D., Tung K.-L., Hsiung C.-C., Chuang C.-J., Ruaan R.-C., Chiang Y.-C., Chen C.-S., Wu T.-H. (2008). Effect of solution chemistry on water softening using charged nanofiltration membranes. Desalination.

[261] Kagramanov G.G., Farnosova E.N., Kandelaki G.I., Václavíková M., Vitale K., Gallios G.P., Ivaničová L. (2010). Heavy Metal Cationic Wastewater Treatment with Membrane Methods. Water Treatment Technologies for the Removal of High-Toxicity Pollutants.

[262] Sudilovskiy P.S., Kagramanov G.G., Trushin A.M., Kolesnikov V.A. (2007). Use of membranes for heavy metal cationic wastewater treatment: Flotation and membrane filtration. Clean Technol. Environ. Policy.

[263] Stamatialis D.F., Papenburg B.J., Gironés M., Saiful S., Bettahalli S.N.M., Schmitmeier S., Wessling M. (2008). Medical applications of membranes: Drug delivery, artificial organs and tissue engineering. J. Membr. Sci..

[264] Gerente C., Lee V.K.C., Cloirec P.L., McKay G. (2007). Application of Chitosan for the Removal of Metals From Wastewaters by Adsorption—Mechanisms and Models Review. Crit. Rev. Environ. Sci. Technol..

[265] Gotoh T., Matsushima K., Kikuchi K. (2004). Preparation of alginate-chitosan hybrid gel beads and adsorption of divalent metal ions. Chemosphere.

[266] Wang W., Zhao Y., Bai H., Zhang T., Ibarra-Galvan V., Song S. (2018). Methylene blue removal from water using the hydrogel beads of poly(vinyl alcohol)-sodium alginate-chitosan-montmorillonite. Carbohydr. Polym..

[267] Petkar K.C., Chavhan S., Kunda N., Saleem I., Somavarapu S., Taylor K.M.G., Sawant K.K. (2018). Development of Novel Octanoyl Chitosan Nanoparticles for Improved Rifampicin Pulmonary Delivery: Optimization by Factorial Design. AAPS Pharmscitech.

[268] Cooper A., Oldinski R., Ma H., Bryers J.D., Zhang M. (2013). Chitosan-based nanofibrous membranes for antibacterial filter applications. Carbohydr. Polym..

[269] Andres Y., Giraud L., Gerente C., le Cloirec P. (2007). Antibacterial effects of chitosan powder: Mechanisms of action. Environ. Technol..

[270] Jagtap S., Yenkie M.K., Das S., Rayalu S. (2011). Synthesis and characterization of lanthanum impregnated chitosan flakes for fluoride removal in water. Desalination.

[271] Beppu M.M., Vieira R.S., Aimoli C.G., Santana C.C. (2007). Crosslinking of chitosan membranes using glutaraldehyde: Effect on ion permeability and water absorption. J. Membr. Sci..

[272] Qi C., Zhao L., Lin Y., Wu D. (2018). Graphene oxide/chitosan sponge as a novel filtering material for the removal of dye from water. J. Colloid Interface Sci..

[273] Amaike M., Senoo Y., Yamamoto H. (1998). Sphere, honeycomb, regularly spaced droplet and fiber structures of polyion complexes of chitosan and gellan. Macromol. Rapid Commun..

[274] Min L.L., Yang L.M., Wu R.X., Zhong L.B., Yuan Z.H., Zheng Y.M. (2019). Enhanced adsorption of arsenite from aqueous solution by an iron-doped electrospun chitosan nanofiber mat: Preparation, characterization and performance. J. Colloid Interface Sci..

[275] Chiou M.-S., Li H.Y. (2002). Equilibrium and kinetic modeling of adsorption ofreactive dye on cross-linked chitosan beads. J. Hazard. Mater. B.

[276] Aliabadi M., Irani M., Ismaeili J., Piri H., Parnian M.J. (2013). Electrospun nanofiber membrane of PEO/Chitosan for the adsorption of nickel, cadmium, lead and copper ions from aqueous solution. Chem. Eng. J..

[277] Aliabadi M., Irani M., Ismaeili J., Najafzadeh S. (2014). Design and evaluation of chitosan/hydroxyapatite composite nanofiber membrane for the removal of heavy metal ions from aqueous solution. J. Taiwan Inst. Chem. E.

[278] Haider S., Park S.-Y. (2009). Preparation of the electrospun chitosan nanofibers and their applications to the adsorption of Cu(II) and Pb(II) ions from an aqueous solution. J. Membr. Sci..

[279] Gupta N., Kushwaha A.K., Chattopadhyaya M.C. (2012). Adsorptive removal of Pb^2+^, Co^2+^ and Ni^2+^ by hydroxyapatite/chitosan composite from aqueous solution. J. Taiwan Inst. Chem. E.

[280] Chen A.-H., Liu S.-C., Chen C.-Y., Chen C.-Y. (2008). Comparative adsorption of Cu(II), Zn(II), and Pb(II) ions in aqueous solution on the crosslinked chitosan with epichlorohydrin. J. Hazard. Mater..

[281] Vakili M., Rafatullah M., Salamatinia B., Abdullah A.Z., Ibrahim M.H., Tan K.B., Gholami Z., Amouzgar P. (2014). Application of chitosan and its derivatives as adsorbents for dye removal from water and wastewater: A review. Carbohydr. Polym..

[282] Li M., Hong Y., Wang Z., Chen S., Gao M., Kwok R.T.K., Qin W., Lam J.W.Y., Zheng Q., Tang B.Z. (2013). Fabrication of Chitosan Nanoparticles with Aggregation-Induced Emission Characteristics and Their Applications in Long-Term Live Cell Imaging. Macromol. Rapid Commun..

[283] Jiang W., Wang W., Pan B., Zhang Q., Zhang W., Lv L. (2014). Facile Fabrication of Magnetic Chitosan Beads of Fast Kinetics and High Capacity for Copper Removal. ACS Appl. Mater. Inter..

[284] Jaiswal A., Ghsoh S.S., Chattopadhyay A. (2012). Quantum Dot Impregnated-Chitosan Film for Heavy Metal Ion Sensing and Removal. Langmuir.

[285] Nayak V., Jyothi M.S., Balakrishna R.G., Padaki M., Ismail A.F. (2015). Preparation and Characterization of Chitosan Thin Films on Mixed-Matrix Membranes for Complete Removal of Chromium. Chemistryopen.

[286] Singh P., Chauhan K., Priya V., Singhal R.K. (2016). A greener approach for impressive removal of As(iii)/As(v) from an ultra-low concentration using a highly efficient chitosan thiomer as a new adsorbent. RSC Adv..

[287] Tang X., Gan L., Duan Y., Sun Y., Zhang Y., Zhang Z. (2017). A novel Cd^2+^-imprinted chitosan-based composite membrane for Cd^2+^ removal from aqueous solution. Mater. Lett..

[288] Gedam A.H., Dongre R.S. (2015). Adsorption characterization of Pb(ii) ions onto iodate doped chitosan composite: Equilibrium and kinetic studies. RSC Adv..

[289] Lei Y., Chen W., Lu B., Ke Q.-F., Guo Y.-P. (2015). Bioinspired fabrication and lead adsorption property of nano-hydroxyapatite/chitosan porous materials. RSC Adv..

[290] Wang X., Li Y., Li H., Yang C. (2016). Chitosan membrane adsorber for low concentration copper ion removal. Carbohydr. Polym..

[291] Razzaz A., Ghorban S., Hosayni L., Irani M., Aliabadi M. (2016). Chitosan nanofibers functionalized by TiO_2_ nanoparticles for the removal of heavy metal ions. J. Taiwan Inst. Chem. E.

[292] Habiba U., Afifi A.M., Salleh A., Ang B.C. (2017). Chitosan/(polyvinyl alcohol)/zeolite electrospun composite nanofibrous membrane for adsorption of Cr^6+^, Fe^3+^ and Ni^2+^. J. Hazard. Mater..

[293] Zavareh S., Behrouzi Z., Avanes A. (2017). Cu (II) binded chitosan/Fe_3_O_4_ nanocomomposite as a new biosorbent for efficient and selective removal of phosphate. Int. J. Biol. Macromol..

[294] Zuo X., Balasubramanian R. (2013). Evaluation of a novel chitosan polymer-based adsorbent for the removal of chromium (III) in aqueous solutions. Carbohydr. Polym..

[295] Mohseni-Bandpi A., Kakavandi B., Kalantary R.R., Azari A., Keramati A. (2015). Development of a novel magnetite–chitosan composite for the removal of fluoride from drinking water: Adsorption modeling and optimization. RSC Adv..

[296] Makaremi M., Lim C.X., Pasbakhsh P., Lee S.M., Goh K.L., Chang H., Chan E.S. (2016). Electrospun functionalized polyacrylonitrile–chitosan Bi-layer membranes for water filtration applications. RSC Adv..

[297] Sabourian V., Ebrahimi A., Naseri F., Irani M., Rahimi A. (2016). Fabrication of chitosan/silica nanofibrous adsorbent functionalized with amine groups for the removal of Ni(ii), Cu(ii) and Pb(ii) from aqueous solutions: Batch and column studies. RSC Adv..

[298] Gokila S., Gomathi T., Sudha P.N., Anil S. (2017). Removal of the heavy metal ion chromiuim(VI) using Chitosan and Alginate nanocomposites. Int. J. Biol. Macromol..

[299] Kousalya G.N., Gandhi M.R., Meenakshi S. (2010). Sorption of chromium(VI) using modified forms of chitosan beads. Int. J. Biol. Macromol..

[300] Gogoi P., Thakur A.J., Devi R.R., Das B., Maji T.K. (2016). A comparative study on sorption of arsenate ions from water by crosslinked chitosan and crosslinked chitosan/MMT nanocomposite. J. Environ. Chem. Eng..

[301] Gupta A., Chauhan V.S., Sankararamakrishnan N. (2009). Preparation and evaluation of iron–chitosan composites for removal of As(III) and As(V) from arsenic contaminated real life groundwater. Water Res..

[302] Bertoni F.A., González J.C., García S.I., Sala L.F., Bellú S.E. (2018). Application of chitosan in removal of molybdate ions from contaminated water and groundwater. Carbohydr. Polym..

[303] Gavilan K.C., Pestov A.V., Garcia H.M., Yatluk Y., Roussy J., Guibal E. (2009). Mercury sorption on a thiocarbamoyl derivative of chitosan. J. Hazard. Mater..

[304] Zainal Z., Hui L.K., Hussein M.Z., Abdullah A.H., Hamadneh I.R. (2009). Characterization of TiO_2_–Chitosan/Glass photocatalyst for the removal of a monoazo dye via photodegradation–adsorption process. J. Hazard. Mater..

[305] Thakre D., Jagtap S., Sakhare N., Labhsetwar N., Meshram S., Rayalu S. (2010). Chitosan based mesoporous Ti–Al binary metal oxide supported beads for defluoridation of water. Chem. Eng. J..

[306] Jóźwiak T., Filipkowska U., Szymczyk P., Kuczajowska-Zadrożna M., Mielcarek A. (2017). The use of cross-linked chitosan beads for nutrients (nitrate and orthophosphate) removal from a mixture of P-PO_4_, N-NO_2_ and N-NO_3_. Int. J. Biol. Macromol..

[307] Jóźwiak T., Filipkowska U., Szymczyk P., Mielcarek A. (2019). Sorption of nutrients (orthophosphate, nitrate III and V) in an equimolar mixture of P–PO_4_, N–NO_2_ and N–NO_3_ using chitosan. Arab. J. Chem..

[308] Arayne M.S., Sultana N., Hussain F. (2011). Validated RP-HPLC Method for Determination of Permethrin in Bulk and Topical Preparations Using UV-vis Detector. J. Chromatogr. Sci..

[309] Rahmanifar B., Dehaghi S.M. (2014). Removal of organochlorine pesticides by chitosan loaded with silver oxide nanoparticles from water. Clean Technol. Environ. Policy.

[310] Saifuddin N., Nian C., Zhan L., Ning K. (2011). Chitosan-silver nanoparticles composite as point-of-use drinking water filtration system for household to remove pesticides in water. Asian J. Biochem..

[311] Danalıoğlu S.T., Bayazit Ş.S., Kuyumcu Ö.K., Salam M.A. (2017). Efficient removal of antibiotics by a novel magnetic adsorbent: Magnetic activated carbon/chitosan (MACC) nanocomposite. J. Mol. Liq..

[312] Nadavala S.K., Swayampakula K., Boddu V.M., Abburi K. (2009). Biosorption of phenol and o-chlorophenol from aqueous solutions on to chitosan-calcium alginate blended beads. J. Hazard. Mat..

[313] Ummadisingu A., Gupta S. (2012). Characteristics and kinetic study of chitosan prepared from seafood industry waste for oil spills cleanup. Desalination Water Treat..

[314] Bibi S., Yasin T., Hassan S., Riaz M., Nawaz M. (2015). Chitosan/CNTs green nanocomposite membrane: Synthesis, swelling and polyaromatic hydrocarbons removal. Mater. Sci. Eng. C.

[315] Fan L., Luo C., Li X., Lu F., Qiu H., Sun M. (2012). Fabrication of novel magnetic chitosan grafted with graphene oxide to enhance adsorption properties for methyl blue. J. Hazard. Mater..

[316] Khan S.B., Ali F., Akhtar K. (2019). Chitosan nanocomposite fibers supported copper nanoparticles based perceptive sensor and active catalyst for nitrophenol in real water. Carbohydr. Polym..

[317] Herman S., Saputra E., Siregar M.R., Panjaitan D.N.I., Pinem J.A. (2019). Synthesis and Characterization of Chitosan-Silica Membranes for Treating Hotel Wastewater Treatment as Affected by Mass of Poly Ethylene Glycol and Poly Vinyl Alcohol. J. Appl. Mater. Technol..

[318] Rosdi N., Sokri M.N.M., Rashid N.M., Chik M.S.C., Musa M.S. (2018). Chitosan/Silica Composite Membrane: Adsorption of Lead(II) Ion from Aqueous Solution. J. Appl. Membr. Sci. Technol..

[319] Truong T., Takaomi K., Bui H. (2019). Chitosan/zeolite composite membranes for the eliminating of trace metal ions in the evacuation permeability process. J. Serb. Chem. Soc..

[320] Sashiwa H., Aiba S.-i. (2004). Chemically modified chitin and chitosan as biomaterials. Prog. Polym. Sci..

[321] da Silva Alves D.C., Healy B., Pinto L.A., Cadaval T.R., Breslin C.B. (2021). Recent Developments in Chitosan-Based Adsorbents for the Removal of Pollutants from Aqueous Environments. Molecules.

[322] Ullah I. (2019). Carbon nanotube membranes for water purification: Developments, challenges, and prospects for the future. Sep. Purif. Technol..

